# Supplementary material for: Co-induction of stromal and epithelial progenitors for renal regeneration
Source: Innovation (Camb). 2026 Jan 29;7(5):101281. doi: 10.1016/j.xinn.2026.101281 (PMC13147989; doi:10.1016/j.xinn.2026.101281)
Supplement: Document S1. Figures S1–S4 and Tables S1 and S2 [file mmc1.pdf]

The Innovation, Volume 7

Supplemental Information

Co-induction of stromal and epithelial progenitors for renal regeneration

Thomas Vincent, Samera Nademi, Michael Namestnikov, Osnat Cohen-Zontag, Benjamin Dekel, and Benjamin S. Freedman

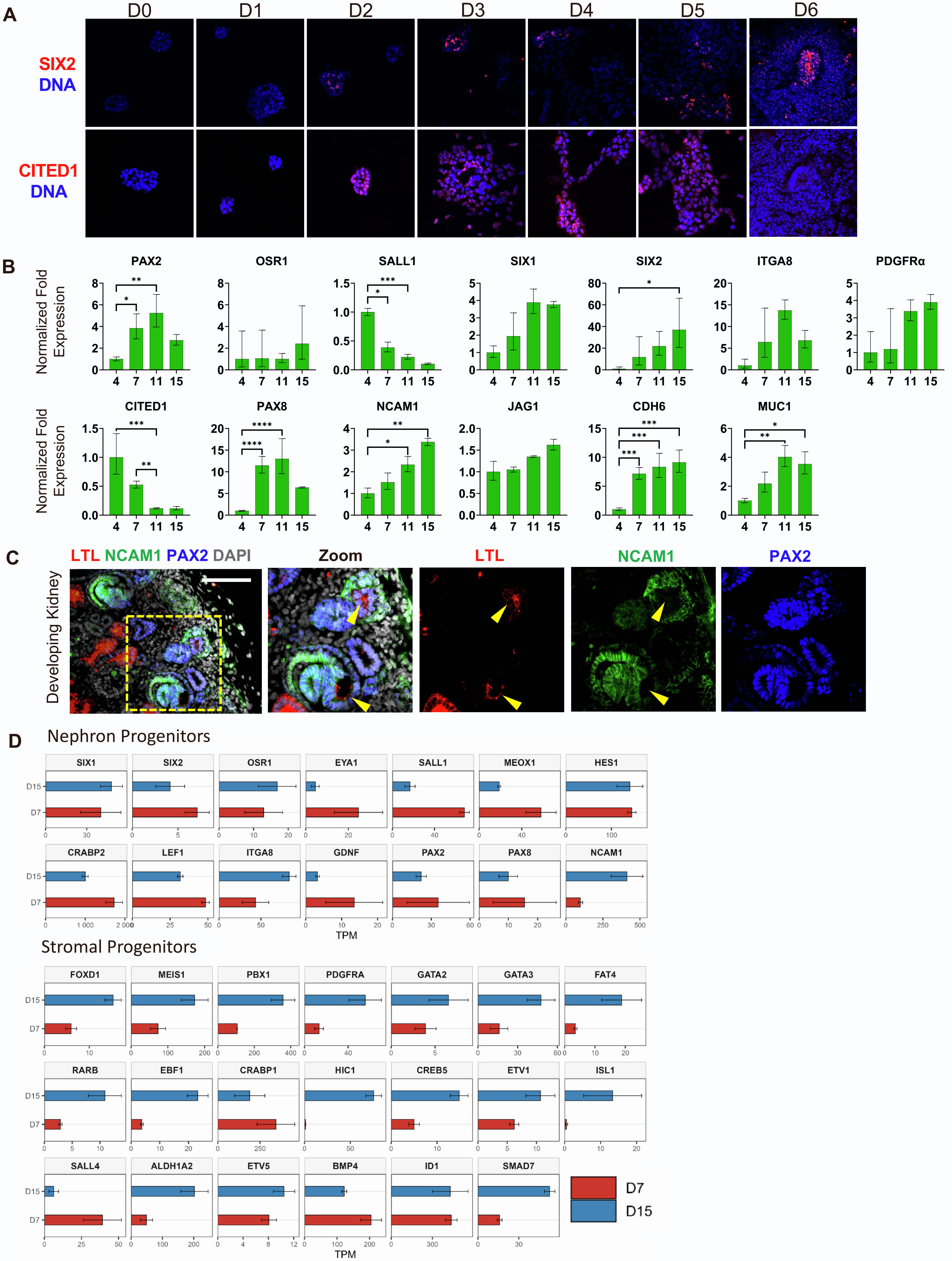
A
D0
D1
D2
D3
D4
D5
D6
SIX2
DNA
CITED1
DNA
B
Normalized Fold Expression
PAX2
OSR1
SALL1
SIX1
SIX2
ITGA8
PDGFRα
CITED1
PAX8
NCAM1
JAG1
CDH6
MUC1
4 7 11 15
C
LTL NCAM1 PAX2 DAPI
Zoom
LTL
NCAM1
PAX2
Developing Kidney
D
Nephron Progenitors
SIX1
SIX2
OSR1
EYA1
SALL1
MEOX1
HES1
CRABP2
LEF1
ITGA8
GDNF
PAX2
PAX8
NCAM1
D15
D7
TPM
Stromal Progenitors
FOXD1
MEIS1
PBX1
PDGFRA
GATA2
GATA3
FAT4
RARB
EBF1
CRABP1
HIC1
CREB5
ETV1
ISL1
SALL4
ALDH1A2
ETV5
BMP4
ID1
SMAD7
D7
D15
TPM

Supplemental Figure 1. qPCR and immunofluorescence depict temporal changes in cell markers in organoids. (A) Representative confocal immunofluorescence images of organoid differentiations from day 0 to day 6 showing increase in signal of NPC marker, SIX2, and an eventual decrease in CITED1. **(B)** qPCR graphs of commonly used kidney differentiation markers on days 4, 7, 11, and 15 where expression has been reported as a fold change in reference to day 0 (mean ± s.e.m. from n = 3 independent biological replicates, * $p < 0.05$, ** $p < 0.01$, *** $p < 0.001$, **** $p < 0.0001$). **(C)** Representative immunofluorescence image of 20-week-old developing human kidney stained for LTL (red), NCAM1 (green), PAX2 (blue), and DAPI (grey). Differentiating structures express LTL as they lose NCAM1 signal (yellow arrowheads). Scale bars = 100 μm (A, C). **(D)** Bulk RNA-seq gene expression levels of nephron progenitor (NP) and stromal progenitor (SP) markers at day 7 (red) and day 15 (blue) as measured by TPM (transcripts per million, mean ± s.e.m.).

Supplemental Figure 2. Induced metanephric mesenchyme forms superior grafts than differentiated organoids.

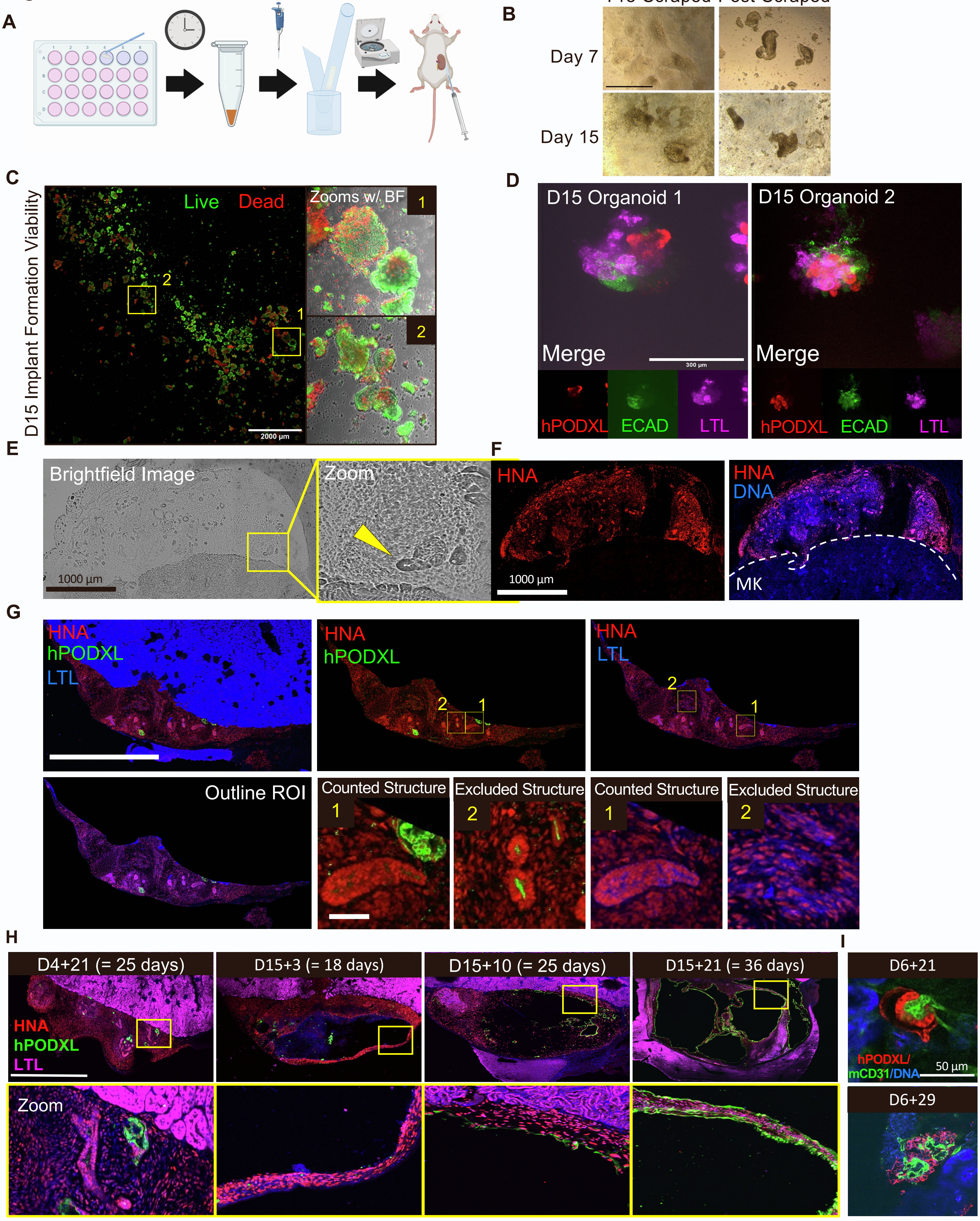

Supplemental Figure 2. Induced metanephric mesenchyme forms superior grafts than differentiated organoids. (A) Schematic depicting implant formation process. **(B)** Example brightfield images of day 7 progenitor cell clusters and day 15 organoids pre- and post- manual dissociation from adherent cultures. Scale bar = 1000 µm. **(C)** Viability staining of organoids that have undergone the implant formation process. Live-Dead analysis performed using Calcein AM and propidium iodide. Green cells take in Calcein AM and are viable, red cells have absorbed PI and are dead. Scale bar = 2000 µm. **(D)** Immunofluorescent images of D15 kidney organoids in suspension that have undergone the implant formation process and dispensed into a well for fixation and staining. **(E)** Representative brightfield image of kidney tissue section. Epithelial structures (tubules and glomeruli) appear darker within the grafts (yellow arrow). Scale bar = 1000 µm. **(F)** Representative confocal immunofluorescent image of graft identification using Human Nuclear Antigen (HNA). Dashed white line depicts border between the graft and the host murine kidney (MK) cortex. Scale bar = 1000 µm. **(G)** Representative images depicting typical workflow process for assessing how many PODXL$^+$ and LTL$^+$ structures are present in graft sections representing glomerular and tubule structures respectively. **(H)** Representative confocal immunofluorescence images of early (day 4) and mature (day 15) organoids implanted beneath the kidney capsule for 3, 10, or 21 days. D4 + 21 (25 days) denotes the combined duration of in vitro organoid culture and in vivo implantation, with the total duration provided in parentheses. **(I)** Representative immunofluorescence images of grafts from day 6 organoids implanted under the kidney capsule for 21 or 29 days. Scale bars = 1000 µm and 50 µm for zoomed panels.

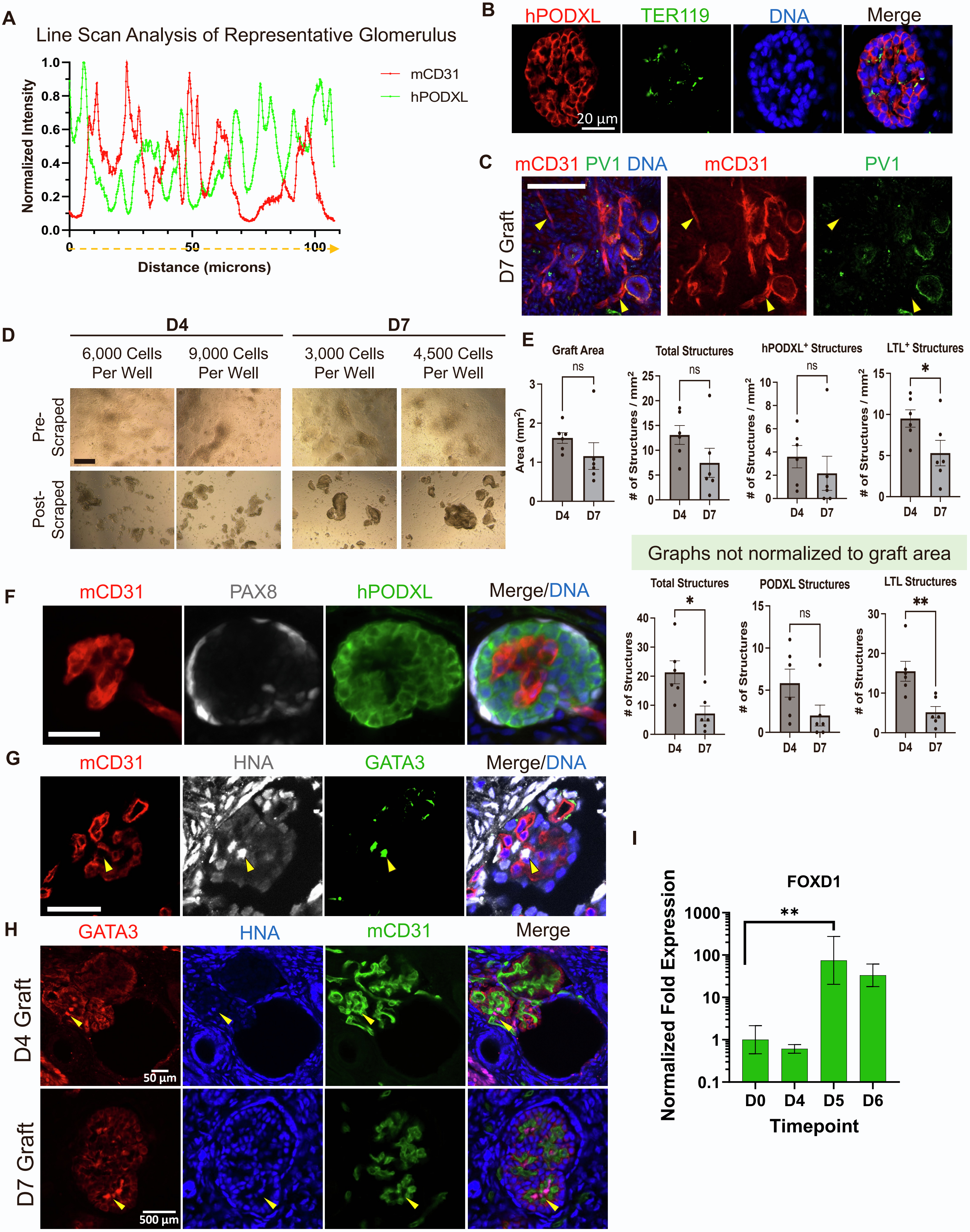
A
Line Scan Analysis of Representative Glomerulus
Normalized Intensity
Distance (microns)
mCD31
hPODXL
B
hPODXL
TER119
DNA
Merge
20 µm
C
mCD31 PV1 DNA
mCD31
PV1
D7 Graft
D
D4
D7
6,000 Cells Per Well
9,000 Cells Per Well
3,000 Cells Per Well
4,500 Cells Per Well
Pre-Scraped
Post-Scraped
E
Graft Area
Total Structures
hPODXL+ Structures
LTL+ Structures
Area (mm2)
# of Structures / mm2
Graphs not normalized to graft area
Total Structures
PODXL Structures
LTL Structures
# of Structures
F
mCD31
PAX8
hPODXL
Merge/DNA
G
mCD31
HNA
GATA3
Merge/DNA
H
GATA3
HNA
mCD31
Merge
D4 Graft
D7 Graft
50 µm
500 µm
I
FOXD1
Normalized Fold Expression
Timepoint
D0
D4
D5
D6

Supplemental Figure 3. iMM can be implanted at very early timepoints of differentiation. (A) Line scan of fluorescence signal (normalized to the max signal of each channel) of the representative chimeric glomerular structure shown in Figure 3A. **(B)** Representative confocal images showing TER119, a mouse red blood cell marker. $TER119^+$ cells are closely associated with human podocytes ($hPODXL^+$), forming chimeric mouse–human capillary-like structures. $TER119^+$ cells are $DAPI^-$, consistent with their enucleated state (n = 3 mice). **(C)** Confocal image of invading vasculature (mCD31) within the graft showing a lack of PV1 expression (yellow arrows). Scale bar = 100 µm. **(D)** Representative brightfield images of starting cell implant material. Scale bar = 500 µm. **(E)** Quantification of total $hPODXL^+$ and LTL^+ structures in D4 and D7 grafts (p-value = 0.1392) as well as cross sectional area (p-value = 0.2369) of representative graft sections. (mean ± s.e.m. from n = 6 independent implantations per time point) **(F)** Representative confocal immunofluorescent images of D4 grafts containing major components of chimeric glomeruli including human podocytes (hPODXL) and parietal epithelial cells (PAX8) and mouse-derived vasculature (mCD31). Scale bar = 25 µm. **(G)** Representative confocal image of $GATA3^+/HNA^+$ human mesangial cells (yellow arrow) within chimeric glomerular structures. Scale bar = 25 µm. **(H)** Representative confocal images of day 4 and day 7 organoid grafts implanted beneath the kidney capsule for 21 days. **(I)** qPCR graph of *FOXD1* where expression has been reported as a fold change in reference to day 0 (mean ± s.e.m. from n = 3 independent biological replicates, * $p < 0.05$, ** $p < 0.01$, *** $p < 0.001$, **** $p < 0.0001$).

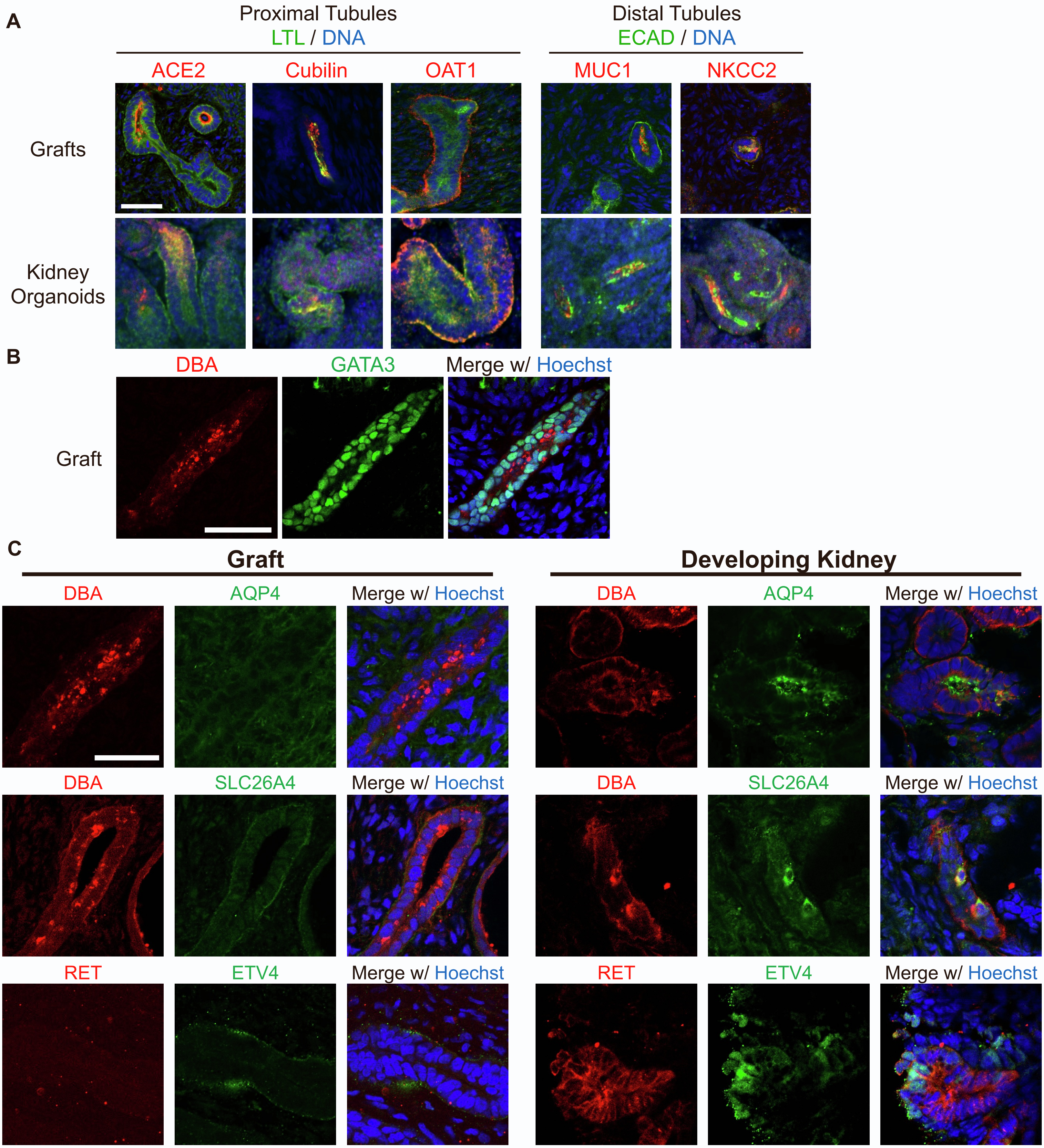

Supplemental Figure 4. Grafts exhibit proximal and distal tubular markers without collecting ducts. (A) Representative immunofluorescent staining in grafts vs. organoids for a panel of markers found in proximal tubules (left, with LTL) and distal tubules (right, with ECAD). (B) Representative immunofluorescent image of graft tubule staining positive for distal markers *Dolichos biflorus* agglutinin (DBA) and GATA3. (C) Representative immunofluorescent images of tubules in grafts (left) and tubules in human developing kidney tissue samples (right) for collecting duct- and ureteric bud-specific markers. Scale bars = 50 µm (A, B) and 25 µm (C).

Supplemental Table 1. List of primers used for RT-qPCR analysis.

Gene	Forward Primer	Reverse Primer
CDH6	CCAGTGGCTCAAACTTTACC	AATGACCACAGGCAAGAGATA
CITED1	AGGATGCCAACCAAGAGATG	GTTTAGTGGGAGGGGTGGTT
CUBN	AATGGATGTGTGCAGCTCAG	GGGGTTGCTCAAACACTCAT
EPCAM	TCTCATCGCAGTCAGGATCATAA	AATCGTCAATGCCAGTGTACTT
FOXD1	GCTCGAGGAAGAAGGTAGGAATC	GCATAGGTCGGCTTTGCATAAAT
HNF1β	GCGCTCAACACCGAGGAG	CAGCAGCTGATCCTGACTGC
ITGA8	GTGCAGTGGGACGACTCGAA	ACACCAGGGATGCAAGAGCATAG
JAG1	TGCCAAGTGCCAGGAAGT	GCCCCATCTGGTATCACACT
LHX1	GGAGCGAAGGATGAAACAGC	TGCGGGAAGAAGTCGTAGTT
LRP2	GCAGACCTAAAGGAGCGTTCG	ATGCGCACTGTCACATTCTTG
MUC1	TCCTCACAGTGCTTACAGTTGTT	ACAGGAAAAAGAAAGAGACCCCA
NCAM1	AACAAAGCATGATGGGTGAA	GTCTGTGGTGTTGGAAATGC
NPHS1	GATCCTCTCCATCCTGGTTCCT	CTCAGGAGAATGGTGATGTCAGG
OSR1	GACATCTGCCACAAAGCCTTC	CCCACAGGTTCTATTTAGCATTTGA
PAX2	AACGACAGAACCCGACTATG	ATCCCACTGGGTCATTGGAG
PAX8	CAAGGTGGTGGAGAAGATTG	GAGGTTGAATGGTTGCTG
PDGFRα	GTGCGAAGACTGAGCCAGATTG	CGATAAACAGAATGCTTGAGCTGTG
PODXL	CAGACCGTGGTCGTCAAAGA	TTCATGTCACTGACCCCTGC
SALL1	TGCAAACAGAATGCAAGCGTTA	AGGCATTGCATCAACACCAGA
SIX1	GGTTTAAGAACCGGAGGCAAAG	AGGACCGAGTTCTGGTCTGGA
SIX2	ACAGGTCAGCAACTGGTTCAAGA	ACTTGCCGCTGCCATTCA

Supplemental Table 2. List of antibodies used for immunofluorescence analysis.

Antibody	Source	Reference	Dilution
ACE2	R&D Systems	Cat. No. AF933	1:40
AQP1	MilliporeSigma	Cat. No. AB2219	1:250
AQP4	Santa Cruz Biotechnology	Cat. No. sc-32739	1:50
hCD31-PE	Miltenyi Biotech	Cat. No. 130-117-225	1:500
mCD31	BD Biosciences	Cat. No. 557355	1:100
CITED1	Invitrogen	Cat. No. PA5-65541	1:300
Cubilin	Abcam	Cat. No. 191073	1:500
DBA	Vector Labs	Cat. No. RL-1032	1:200
ECAD	Abcam	Cat. No. ab11512	1:300
ETV4	ProteinTech	Cat. No. 10684-1-AP	1:100
GATA3	R&D Systems	Cat. No. AF2605	1:100
HNA	EMD Millipore	Cat. No. MAB1281	1:300
Pan-Laminin	Abcam	Cat. No. ab11575	1:300
LTL	Vector Labs	Cat. No. B-1325	1:500
MEIS 1/2/3	Active Motif	Cat. No. 39795	1:1000
MUC1	Abcam	Cat. No. ab15481	1:200
NCAM	Santa Cruz	Cat. No. sc-106	1:250
NKCC2	ProteinTech	Cat. No. 18970-1-AP	1:500
OAT1	Invitrogen	Cat. No. PA5-26244	1:500
PAX2	BioLegend	Cat. No. 901001	1:250
PAX8	ProteinTech	Cat. No. 10336-1-AP	1:100
PDGFRβ	R&D Systems	Cat. No. AF385	1:100
PV1	Abcam	Cat. No. ab81719	1:100
hPODXL	R&D Systems	Cat. No. AF1658	1:500
RET	R&D Systems	Cat. No. AF1485	1:300
SIX1	Cell Signaling Technology	Cat. No. 12891	1:1000
SIX2	ProteinTech	Cat. No. 11562-1-AP	1:300
SLC26A4	Sigma	Cat. No. SAB2104723	1:500
UMOD	Sigma	Cat. No. SAB1400296	1:100
