# Supplementary material for: Co-induction of stromal and epithelial progenitors for renal regeneration
Source: Innovation (Camb). 2026 Jan 29;7(5):101281. doi: 10.1016/j.xinn.2026.101281 (PMC13147989; doi:10.1016/j.xinn.2026.101281)
Supplement: Document S2. Article plus supplemental information [file mmc2.pdf]

REPORT Basic Medicine

OPEN ACCESS

Co-induction of stromal and epithelial progenitors for renal regeneration

Thomas Vincent,[1,2,3] Samera Nademi,[1,2] Michael Namestnikov,[4,5,6] Osnat Cohen-Zontag,[5,6] Benjamin Dekel,[4,5,6,*] and Benjamin S. Freedman[1,2,3,7,8,*]
*Correspondence: benjamin.dekel@gmail.com (B.D.); benof@uw.edu (B.S.F.)
Received: April 16, 2025; Accepted: January 26, 2026; Published Online: January 29, 2026; https://doi.org/10.1016/j.xinn.2026.101281
© 2026 The Authors. Published by Elsevier Inc. on behalf of Youth Innovation Co., Ltd. This is an open access article under the CC BY license (http://creativecommons.org/licenses/by/4.0/).

GRAPHICAL ABSTRACT

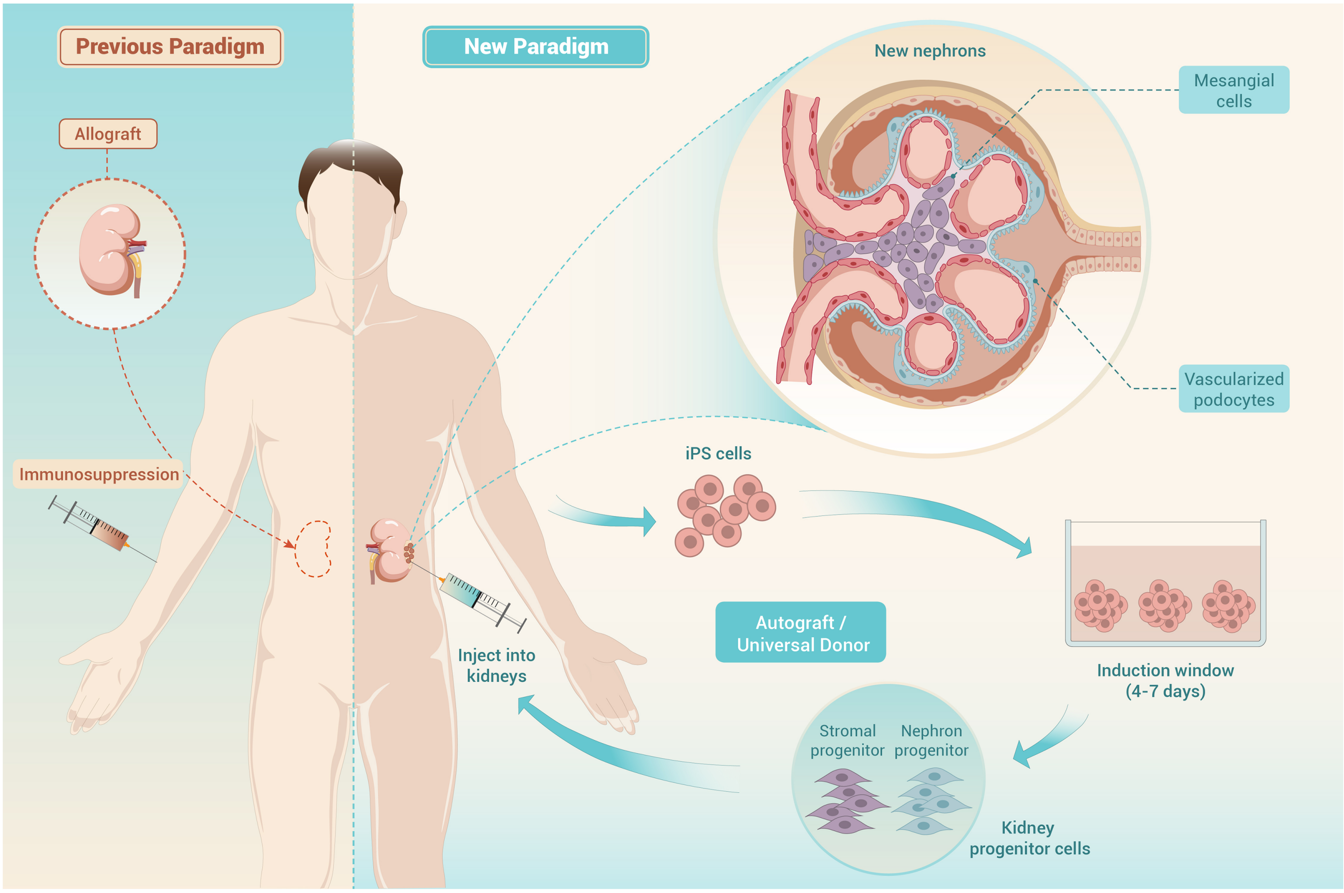

PUBLIC SUMMARY

- Four days of differentiation generates an induced metanephric mesenchyme (iMM).
- iMM contains both nephron progenitor cells and renal stromal progenitor cells.
- iMM gives rise to sophisticated, vascularized nephron-like structures beneath the kidney capsule.
- In contrast to iMM, mature organoids rapidly lose differentiated features and fail to productively engraft.
- iMM grafts contain mesangial cells in addition to mature tubules.

Co-induction of stromal and epithelial progenitors for renal regeneration

Thomas Vincent,[1,2,3] Samera Nademi,[1,2] Michael Namestnikov,[4,5,6] Osnat Cohen-Zontag,[5,6] Benjamin Dekel,[4,5,6,*] and Benjamin S. Freedman[1,2,3,7,8,*]
[1]Division of Nephrology, Department of Medicine, University of Washington School of Medicine, Seattle WA 98109, USA
[2]Institute for Stem Cell and Regenerative Medicine, University of Washington School of Medicine, Seattle, WA 98109, USA
[3]Department of Bioengineering, University of Washington, Seattle, WA 98195, USA
[4]Division of Pediatric Nephrology, Edmond & Lily Safra Children's Hospital, Sheba Medical Center, Tel Hashomer 52621, Israel
[5]Pediatric Stem Cell Research Institute, Edmond & Lily Safra Children's Hospital, Sheba Medical Center, Tel Hashomer 52621, Israel
[6]Sagol Center for Regenerative Medicine, School of Medicine, Faculty of Medicine, Tel Aviv University, Tel Aviv, Israel
[7]Kidney Research Institute, University of Washington, Seattle, WA 98109, USA
[8]Plurexa, 1209 6th Ave. N., Seattle, WA 98109, USA
*Correspondence: benjamin.dekel@gmail.com (B.D.); benof@uw.edu (B.S.F.)
Received: April 16, 2025; Accepted: January 26, 2026; Published Online: January 29, 2026; https://doi.org/10.1016/j.xinn.2026.101281
© 2026 The Authors. Published by Elsevier Inc. on behalf of Youth Innovation Co., Ltd. This is an open access article under the CC BY license (http://creativecommons.org/licenses/by/4.0/).
Citation: Vincent T., Nademi S., Namestnikov M., et al., (2026). Co-induction of stromal and epithelial progenitors for renal regeneration. The Innovation **7(5)**, 101281.

Multiple pools of developmental progenitors are needed for kidney regeneration, but directing differentiation of each of these independently is rate limiting and disruptive to the process. We demonstrate that a mere 4 days of differentiation generates an induced metanephric mesenchyme (iMM) containing both nephron progenitor cells and renal stromal progenitor cells. When implanted beneath the kidney capsule of immunodeficient mice, iMM differentiates into podocytes, mesangial cells, and tubules that recruit host blood vessels to form glomeruli and peritubular capillaries. In contrast, mature organoids rapidly lose differentiated features after implantation and fail to become productively vascularized by the host. By examining morphological changes and gene expression patterns, we demarcate a window of opportunity for successful implantation during which iMM exists *in vitro*. Young organoid cultures containing epithelial and stromal progenitor cells thus provide a rapid, one-pot starting material for nephron regeneration, a concept that may be broadly applicable to many organ systems.

INTRODUCTION

Chronic kidney disease is a major contributor to morbidity and mortality, affecting approximately 14% of the US adult population as of 2022, with 800,000 patients in need of renal replacement therapy.[1] The demand for kidney transplants far outweighs supply, and, as all kidney transplants are allografts, they rarely last longer than ~12 years, even with chronic immunosuppression. Induced pluripotent stem cells (iPSCs) can be generated from any individual and could potentially provide an autologous source of renal tissue on demand.[2] Over the past decade, the first differentiation protocols have been developed to differentiate iPSCs into kidney organoids patterned with major segments of the nephron.[3–6] These protocols commonly feature an early induction step with CHIR99021 (CHIR), an inhibitor of glycogen synthase kinase-3β, which induces iPSCs to transition through a mesenchyme-like progenitor stage before differentiating into segmented epithelial organoids. This process is thought to mimic the process of nephrogenesis, during which varied nephron epithelial cells differentiate from a common nephron progenitor cell (NPC) population that arises within the metanephric mesenchyme (MM).[7]

During renal organogenesis, specialized renal stromal progenitor cells (SPs; also referred to as interstitial progenitor cells) arise within the MM, which subsequently differentiate into glomerular mesangial cells and interstitial populations and facilitate nephron epithelial differentiation via crosstalk mechanisms.[8–10] Human kidney organoids also contain a significant population of stromal cells, which includes endothelial cells, but whether this stroma derives from SPs or represents an "off-target" population of iPSC differentiation is not yet clear.[3,11–13] Glomerular mesangial cells, for instance, which are among the most readily identifiable renal stromal cells *in vivo*, are not readily evident in kidney organoid cultures.[14] One approach to address this deficiency is to differentiate iPSCs into NPCs and SPs separately and then combine these to construct higher-order macrostructures, although this requires careful coordination of differentiation protocols and has not yet been successful with human iPSCs.[14,15]

For renal regeneration, the kidney capsule has emerged as a convenient and conducive site for implantation in which organoids can form glomerulus-like structures vascularized by an immunodeficient host.[16–19] It is not yet known, however, what the ideal stage of differentiation is for productive implantation. Some studies have implanted mature organoids to produce sophisticated glomerulus-like structures,[17,19] whereas other studies suggest limited potential for mature organoids[16] or have implanted earlier-stage NPCs.[18,20] It is also unclear whether these grafts contain any human stromal cells specific to the kidneys.[19] These questions prompted us to conduct a comparative study of graft differentiation state to determine the optimal source material for implantation.

MATERIALS AND METHODS

Experimental model and study participant details

Animals. All animal studies were conducted in accordance with all relevant ethical regulations under protocols approved by the Institutional Animal Care and Use Committee at the University of Washington in Seattle (protocol #4375-01). Non-obese diabetic (NOD)-severe combined immunodeficiency (SCID) mice (Research Resource Identifier [RRID]: IMSR_JAX:001303), 8- to 10-week-old adult males and females were utilized for the implantation experiments. Mice were maintained under standard pathogen-free housing conditions, with *ad libitum* access to food and water.

Cell lines. WTC11 human pluripotent stem cells (hPSCs) (Coriell 25256, female) were fed and passaged on 1% reduced growth factor GelTrex (Life Technologies)-coated tissue culture plates in mTeSR1 medium (Stem Cell Technologies) and dissociated into single cells using Accutase (Stem Cell Technologies).

Human kidney samples. Any discarded human kidney samples used for reference were obtained under the approval of the University of Washington Institutional Review Board.

Method details

Kidney organoid differentiation. Human iPSC stock cell lines were maintained in adherent cultures in six-well plates with daily changes of mTeSR1 medium (STEMCELL Technologies) and weekly or bi-weekly passaging using Accutase (STEMCELL Technologies) or ReLeSR (STEMCELL Technologies). For differentiations, cells are dissociated to single-cell state with Accutase and seeded onto a 24-well tissue-culture-treated plate pre-coated with 300 μL of DMEM-F12 + 1% GelTrex LDEV-Free (Thermo Fisher Scientific) at a density of 1,000–2,000 cells per well in ≥0.5 mL of mTeSR1 containing 10 μM Rho-Kinase inhibitor Y27632 (Tocris Biosciences) on day −3. The next day, the medium was replaced with mTeSR1 + 1.5% GelTrex before returning to standard mTeSR1 on day −1 to create isolated spheroid colonies. The differentiation is induced on day 0 by switching the medium to Advanced RPMI + 1% Glutamax (Life Technologies) + 12 μM CHIR99021 (Tocris Bioscience) for 36 h and then RB maintenance medium (Advanced RPMI + 1% Glutamax + 2% B27 Supplement [Life Technologies]). Maintenance medium was then replaced every 2–3 days thereafter so that fresh cell medium was added on the morning of performing any implantation experiment.

qPCR analysis. To prepare RNA, cells were lysed at room temperature by aspirating cell culture medium and replacing it with 500 μL of TRIzol (Thermo Fisher Scientific, 15596018) per well of a 24-well plate (or 75 μL per well of a 96-well plate). Cells were homogenized by scraping the well with the pipet tip and pipetting up and down several times and then freezing them in a −80°C freezer or processing them immediately. Next, 0.2 mL of chloroform was added per 1 mL of TRIzol cell solution, it was left at room temperature

for 2–3 min, and then it was centrifuged for 15 min at 12,000 rpm at 4°C. The colorless, aqueous phase was transferred to a tube containing an equal amount of 70% ethanol and then transferred to a spin cartridge. RNA was further purified by using the Invitrogen TRIzol Plus RNA Purification Kit (Invitrogen). DNase was used to eliminate any residual genomic DNA, samples were measured using a NanoDrop, and purified RNA was stored in a −80°C freezer or processed further.

500–1,000 ng of RNA was reverse transcribed using the SuperScriptIV Reverse Transcription kit (Invitrogen). For each independent experiment, RT-qPCR reactions were run in triplicate using cDNA (diluted 1:15), 300 nM primers, and PowerUp SYBR Green Master Mix (Applied Biosystems). qPCR primer sequences are listed in Table S1. mRNA expression was calculated using the 2^{-DDCT} method, where cycle counts (CTs) of each gene of interest were normalized to a housekeeper gene (dCT) (β-actin or GAPDH) and then the difference in cycles was compared across samples (ddCT). -ddCT was then used as the exponent to calculate the difference in expression levels as fold changes compared to the first point sampled of each series (e.g., D0 or D4). See Table S1 for a list of primers.

RNA sequencing data analysis. A recently published dataset from our group including samples of iPSC-derived kidney organoids on days 7 or 15 after induction with CHIR99021 (days 10 or 18 after plating) was re-analyzed using the methods described.[21] Markers of NPCs and SPs were selected based on prior datasets.[15,21] The R packages pheatmap and ggplot2 were used for data visualization.

Renal progenitor cell and kidney organoid implant formation. 2–4 wells of a 24-well plate are selected for each implant based on visual observation under a brightfield microscope. Medium in each well is replaced with 300 μL of fresh medium and a mini cell lifter is used to manually scrape the cells out of the well. Cells are collected in a microcentrifuge tube and allowed to settle to the bottom of the tube for at least 15 min in an incubator. The condensed material is then taken up into a 200-μL micropipette, briefly allowed to settle to the tip, and then dispensed into PE50 polyethylene tubing with a bevel on one side. This tubing is then kinked on one end to prevent material from escaping and centrifuged at 300 *g* for 4 min to aggregate the pellet.

Kidney subcapsular implantation surgery, excision, and processing. The cell pellets are implanted beneath the kidney capsule of mice. The procedure is based on the protocol from Szot et al.[22] The kidneys are levered outside of the abdomen through a dorsal incision and held in place using a cotton-tipped applicator. The capsule is nicked with a 27-gauge needle and the beveled end of the tubing is inserted and rotated to form a pocket. The pellet is dispensed by dialing down a Hamilton syringe connected at the far end of the tubing and the kidney is blotted with a dry cotton-tipped applicator to promote closure returned to the abdominal cavity. The muscle layer is stitched closed and several staples are used to close the incision. After the specified period of time, the kidney is excised from the mouse, fixed in 4% paraformaldehyde (PFA) for 1.5 h, transferred to 30% sucrose overnight, bisected, and then mounted in Optimal Cutting Temperature Compound (Tissue-Tek) before flash freezing.

For sectioning, cryo blocks were faced and 10- to 20-μm-thick sections were then cut through the kidney and immediately evaluated under a brightfield microscope to identify the location of the graft. Epithelial renal structures in the graft are easily distinguished using a brightfield scope. When locating structures, serial sections were collected and placed on slides so that there was >200 μm between sections on the same slide. If structures were not observed, 100–200 μm was skipped and a new section was cut. This process was repeated through the entire graft.

Immunofluorescence analysis. PFA-fixed cells or frozen slides were blocked in blocking buffer (5% normal donkey serum, 0.3% Triton X-100 in 1× PBS) for 15 or 60 min at room temperature, respectively. Primary antibody incubation was carried out in an antibody dilution buffer (1% BSA, 0.3% Triton X-100 in 1× PBS) overnight at 4°C. Cells were then washed three times for 5 min each in 1× PBS, followed by overnight incubation with secondary antibodies and DAPI/Hoechst nuclei stain diluted in antibody dilution buffer at 4°C. Secondary antibodies were then removed with another round of PBS washes and slides were sealed in PBS. Calcein AM (Invitrogen) and propidium iodide (Thermo Fisher) viability staining was performed according to accompanying product protocols. Images were acquired using a Nikon A1R scope with a point-scanning confocal system or a Yokogawa W1 Spinning Disk confocal head mounted on an inverted Nikon Ti widefield microscope. See Table S2 for an antibody list.

Transmission electron microscopy analysis. Grafts were excised after 3 weeks *in vivo*, dissected into 1-mm cubes, and fixed. Samples were fixed overnight using 4% glutaraldehyde in 0.1 M sodium cacodylate buffer (pH 7.2–7.4) at 4°C. Tissues were then washed 5× for 5 min in buffer at room temperature and post-fixed on ice in buffered 2% osmium tetroxide for 1 h. This was followed by five washes in double-distilled H_2O (dd$H_2$0) and then *en bloc* stained in 1% uranyl acetate (aqueous) overnight at 4°C. The next day, the tissue was washed 5× for 5 min in ddH_2O then dehydrated in progressively increasing ice-cold 30%, 50%, 70%, and 95% EtOH solutions before being allowed to come to room temperature. This was followed by two changes of 100% ETOH and two changes of propylene oxide. The tissue was then infiltrated using a 1:1 mixture of propylene oxide:Epon Araldite resin for 2 h followed by two changes of fresh Epon Araldite for 2 h each. It was then placed in flat embedding molds and polymerized at 60°C overnight. Samples were sectioned at 1- to 2-μm thickness to look for regions of interest in the graft, then ultra-thin sections of 80 nm were cut on a Leica EM UC7 and post-stained with Reynolds Lead Citrate for imaging on a JEOL 1230 at 80KV.

Quantification and statistical analysis

Statistical analyses. To conduct controlled experiments assessing specific variables, we used the same technique for each implant formation and compared grafts of different time points using paired differentiations and pooled the results from multiple rounds of differentiations. Error bars are mean ± standard error (SEM). Statistical analyses were performed using Microsoft Excel and GraphPad Prism software. Details for statistical tests used, how data are displayed, number of replicates for each experiment, and what constituted an independent sample are included in the corresponding figure legends. To test significance, *p* values were calculated using Welch's two-tailed, unpaired *t* test or one-way ANOVAs with Tukey (Dunnett test used to compare FOXD1 expression to the control group at D0) *post hoc* comparisons. For RT-qPCR analysis, statistics were performed on ddCT measurements to get mean ± (SEM) to find significant differences, and then these values were converted to average fold-change expression with upper and lower limits based on the method described in Taylor et al.[23] Statistical significance was defined as $*p < 0.05$, $**p < 0.01$, $***p < 0.001$, and $****p < 0.0001$. Selected exact *p* values are provided in the figure legends in experiments that showed significant results.

Immunofluorescence image analysis and quantification: Number of hPODXL+ and LTL+ structures and line scans. Quantification of on-target structures and line-scan analysis were both performed using FIJI (ImageJ, NIH). For structure quantification, representative sections containing renal structures within the graft were stained with human nuclear antigen (HNA), anti-human podocalyxin (hPODXL), and *Lotus tetragonolobus* lectin (LTL) and a single stitched image was captured. A region of interest (ROI) of the graft was generated based on the HNA staining, and structures of a particular morphology and staining were counted within the section. For grafts where structures were not located by brightfield inspection during sectioning, representative sections of grafts were still stained with HNA, hPODXL, and LTL to confirm the lack of glomeruli and tubules. Each data point represents an individual animal. In some graphs, the number of structures is displayed as #/graft area where the counts were normalized by dividing by the cross-sectional area of the whole graft measured in FIJI.

For line-scan analyses, slides stained with mCD31 and hPODXL were imaged and individual glomerular structures from all day 7 grafts imaged were analyzed (25 total across seven grafts). In FIJI, a line tool was drawn spanning the structures to include the entirety of the outermost podocytes. Lines were drawn perpendicularly to the vascular-urinary pole axis for clarity. The "spline" was set to a width of 10 pixels to get a better representative value of fluorescence measurements of each channel at that position. To average the measurements from different glomeruli, signal intensity for each channel was normalized to the maximum intensity of that channel throughout the line scan, and distance/position was normalized to the total length of the line. Larger glomeruli were down-sampled to the smallest structure to allow for averaging of values. Graphs show mean ± SEM.

RESULTS

Early organoids contain an induced MM population

To define the time points at which NPCs arise in human kidney organoid cultures, we applied an adherent culture protocol involving a single 36-h pulse of CHIR, which induces the subsequent differentiation of tubular organoid structures by day 15 (Figures 1A and 1B).[3] This differentiation protocol is relatively simple, enabling clean separation of the stages of differentiation, compared to other protocols that feature a second CHIR pulse.[4,6,12] Samples for qPCR and immunofluorescent analysis were taken every day starting immediately prior to the CHIR pulse until 4 days after the end of the pulse (D0–D6) (Figures 1C, 1D, and S1A). During the first 7 days of differentiation, genetic markers of intermediate mesoderm (*PAX2*, *OSR1*) were upregulated first, followed by markers of MM (*SALL1*, *SIX1*), NPCs (*SIX1*, *SIX2*, *ITGA8*), and SPs (*PDGFRA*). Comparing earlier to later time points revealed that *SIX2* RNA expression was maintained through D15, whereas *CITED1* expression dropped off after D7 indicating the transition from a self-renewing to a differentiating NPC population (Figure S1B). Markers of epithelialization and nephron segment differentiation, including the tubular markers *EPCAM*, *LHX1*, and *HNF1B*; podocyte markers *PODXL* and *NPHS1*; and tubule maturation markers megalin (*LRP2*)

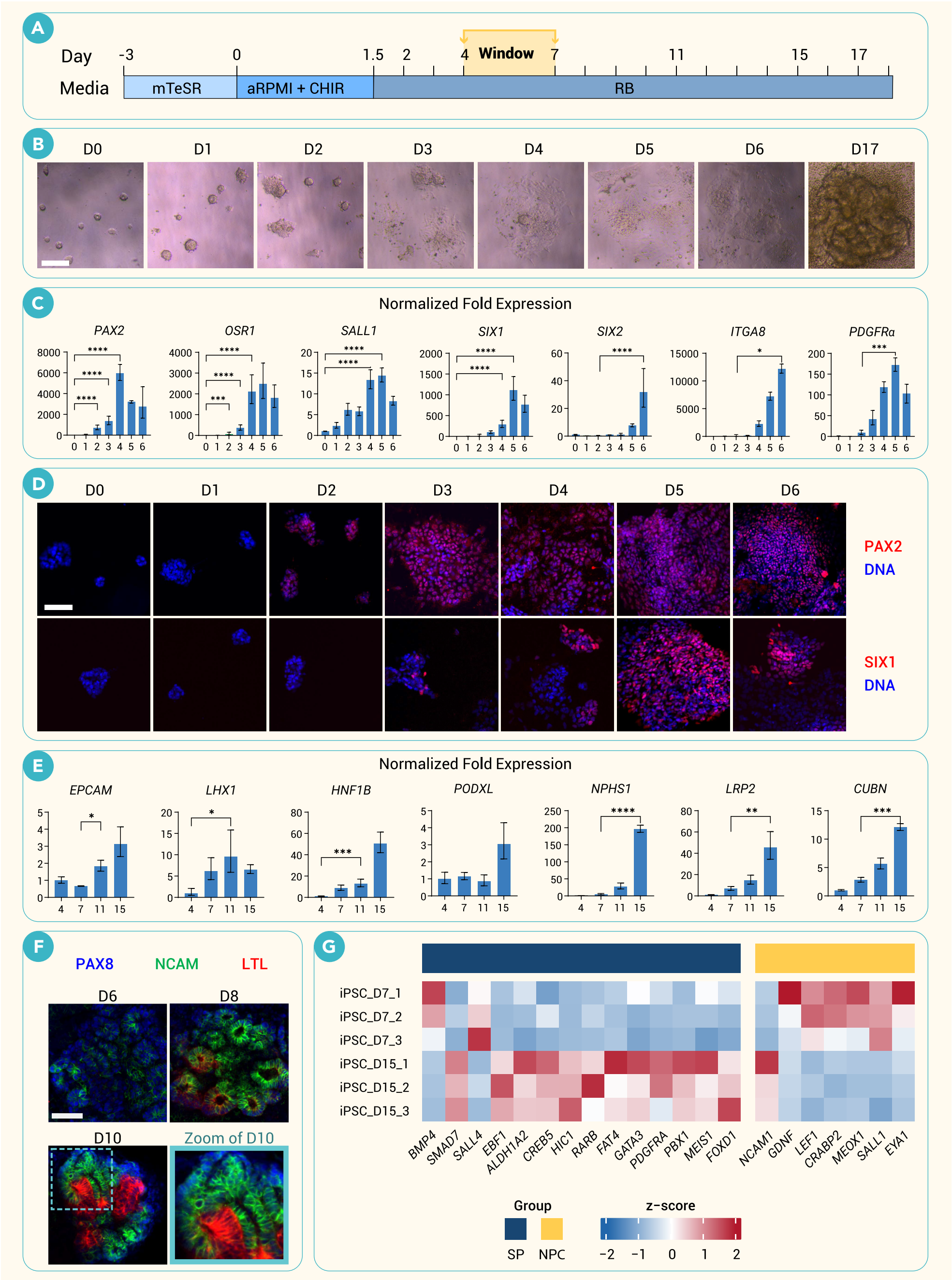

Figure 1. Temporal changes in cell markers indicate a window of opportunity for engraftment (A) Schematic depicting the timeline of differentiation of embryonic spheroids to kidney organoids with stages on the top showing CHIR pulse/culture conditions and bottom showing kidney development stages. The 'window' for optimal engraftment is indicated, based on the results of this study. (B) Representative brightfield images of kidney differentiations from day 0 to day 6 and day 17, where day 0 represents the beginning of the 36-h CHIR pulse. (C) qPCR graphs of commonly used kidney differentiation marker (PAX2, OSR1, SALL1, SIX1, SIX2, ITGA8, and PDGFRα) expression from day 0 to day 6, where expression has been reported as a fold change in reference to day 0. (D) Representative confocal immunofluorescence images of organoid differentiations from day 0 to day 6 showing increase in signal of kidney differentiation markers (PAX2 and SIX1). (E) qPCR graphs of kidney differentiation and maturation markers (EPCAM, LHX1, HNF1β, PODXL, NPHS1, LRP2, and CUBN) on days 4, 7, 11, and 15, where expression has been reported as a fold change in reference to day 0. (F) Representative confocal immunofluorescence images of differentiation days 6, 8, and 10 showing expression patterns of PAX8, NCAM, and LTL. (G) Heatmap showing expression of key signaling molecules and transcription factors in iPSC-derived kidney organoids at days 7 and 15. Scale bars, 200 μm (B), 100 μm (D and F). (qPCR graphs shown as mean ± SEM from n = 3 independent biological replicates, selected significant comparisons shown: *p < 0.05, **p < 0.01, ***p < 0.001, ****p < 0.0001 using a one-way ANOVA with *post hoc* tests).

and cubilin (*CUBN*), increased after D7 (Figure 1E). The NPC marker, neural cell adhesion molecule 1 (NCAM1),[24,25] co-localized with early-forming PAX8$^+$ NPCs in renal vesicle-like rosettes, which by D8 elongated into tubules with affinity for LTL, a marker of differentiated proximal tubules (Figure 1F). This mimicked staining of LTL at the distal end of S-shaped bodies in developing human kidneys (Figure S1C). Re-analysis of a bulk RNA sequencing (RNA-seq) dataset from organoid cultures[21] revealed enrichment of markers of NPCs and stromal progenitors (SPs)[15] on days 7 and 15, respectively (Figures 1G and S1D). Together, these data suggested that an induced MM (iMM) population arises in kidney organoid cultures between D4 and D7, which is followed by NPC differentiation with SP differentiation shortly thereafter.

iMM is superior to differentiated organoids for graft formation

To evaluate the engraftment potential of early (D7) versus late (D15) cultures, implantations beneath the kidney capsule were performed at different time points from the same batches of differentiating organoids (Figures 2A and S2A). In a previous study of implantation using the same differentiation protocol, mature epithelial organoids were purified by microdissection prior to implantation, resulting in only limited vascularization of podocyte clusters.[16] In this study, we used a cell scraper to collect all of the cells within the adherent cultures, including both epithelial and stromal compartments (Figure S2B). Inclusion of stroma was anticipated to potentially enhance organoid maturation after transplant and furthermore enabled direct comparisons to earlier differentiation stages at which microdissection would not be feasible. When examining the impact of this process on the starting material, we observed considerable maintenance of cell viability and maintenance of organoid macrostructure (Figures S2B–S2D).

Implants from both time points were delivered beneath the kidney capsule[3,16,22] and grown *in vivo* for 21 days so that the only difference was the differentiation day of the starting material. Upon excision, opaque tissue masses could be observed beneath the capsule (Figure 2B). When sectioning samples, unstained engrafted tissue containing renal epithelium was easily identifiable by brightfield illumination as the material was lighter than adjacent host kidney cortex, less densely packed, and contained within clearly defined regions beneath the capsule (Figure S2E). HNA further confirmed the presence of human cells within these regions (Figure S2F). Strikingly, early implants contained a greater quantity of podocytes and tubules than late implants (Figure 2C). Quantification of representative sections revealed that early grafts contained approximately a 4-fold increase in nephron structures (hPODXL$^+$ or LTL$^+$) per mm^2 of graft area as compared to D15 grafts (Figure 2D). Only structures that had typical glomerular and tubular morphologies and stained positive for each respective marker were counted (Figure S2G).

We examined the fate of mature organoids after implantation in a time-course analysis. When late organoids were excised after 3, 10, and 21 days *in vivo*, the organization of hPODXL$^+$ podocytes and LTL$^+$ proximal tubules deteriorated in proportion to time post engraftment (Figure 2E). Large cysts were apparent within the graft shortly after implantation, and the grafts appeared viable and proliferative through day 21, as indicated by the increased cross-

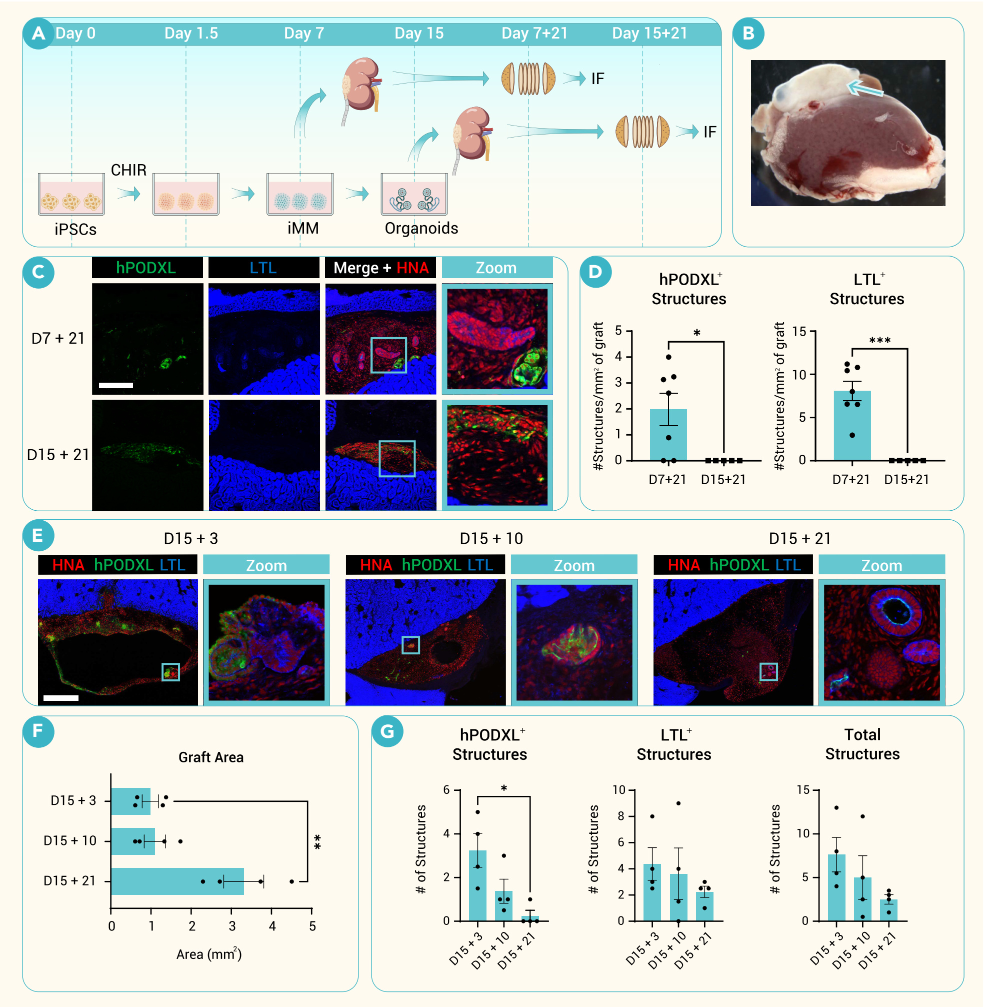

Figure 2. iMM is superior to differentiated organoids for graft formation (A) Schematic of experimental design to compare early, day 7 progenitor cells, to late, day 15 organoids. (B) Stereomicroscope image of excised kidney. D7 subcapsular progenitor cell graft identified by yellow arrow. (C) Representative immunofluorescent HNA/hPODXL/LTL images for D7 and D15 grafts showing lack of structures in the D15 grafts. (D) Quantification of hPODXL$^+$ and LTL$^+$ structures in D7 and D15 grafts (mean ± SEM). D7: n = 7 mice, 1.981 hPODXL$^+$ structures/mm^2, 8.069 LTL$^+$ structures/mm^2. D15: n = 5, 0 hPODXL$^+$ structures/mm^2, 0 LTL$^+$ structures/mm^2. hPODXL p = 0.0193. LTL p = 0.0004. (E) Representative HNA/hPODXL/LTL whole-field immunofluorescent confocal images of representative sections of day 15 grafts after 3, 10, and 21 days *in vivo*. (F) Comparison of cross-sectional graft area of day 15 implants after 3, 10, and 21 days (mean ± SEM, n = 4 mice per group). (G) Quantification of hPODXL$^+$ glomerular structures and LTL$^+$ tubule structures in day 15 grafts (mean ± SEM, n = 4 mice per group). Scale bars, 200 μm (C), 500 μm (E). Selected significant comparisons shown in graphs: *p < 0.05, **p < 0.01, ***p < 0.001, ****p < 0.0001 using Welch's t tests or one-way ANOVA.

sectional area of representative sections imaged (Figure 2F). On day 3 after implantation, the cysts were largely lined by stromal cells, but, by day 10, they had begun to be lined with cells expressing hPODXL, likely reflecting outgrowth of nephron epithelial cells into the cystic spaces (Figure S2H). Counting the absolute number of renal-specific cell clusters revealed a significant loss of podocytes in glomerulus-like structures by day 21 (Figure 2G).

In contrast to grafts of mature organoids (15 days *in vitro* + 10 days *in vivo*), grafts of iMM (4 days *in vitro* + 21 days *in vivo*) did not exhibit substantial cysts, even though the total growth period of 25 days was equivalent (Figure S2H). In addition, iMM grafts maintained glomerular structures even 29 days after implantation (Figure S2I). Together, these data showed that early cultures containing iMM formed superior nephron-like grafts *in vivo* to late cultures containing terminally differentiated organoids, which undergo a cystic degradation process.

iMM grafts form glomeruli with human mesangial cells

Strikingly, iMM grafts formed intricate glomerulus-like structures (hitherto, glomeruli) encompassing a murine capillary tuft expressing mouse-specific CD31 (mCD31) enveloped by human podocytes (hPODXL$^+$) and parietal epithelial cells (PECs, PAX8$^+$) (Figures 3A–3C). Line-scan analysis through individual glomeruli perpendicular to the vascular-urinary pole axis revealed increased hPODXL signal on the exterior of these structures and increased mCD31 in the interior, reflecting encapsulation of the vasculature by the podocytes (Figures 3B and S3A).

The sophisticated morphology of these glomeruli, including contorted vascular beds, suggested that mesangial cells may be present.[26] Indeed, mesangial cells were identified within glomeruli by bright, compact GATA3$^+$ nuclei on the opposite side of the capillaries from the podocytes, which co-localized with HNA, indicating they were human in origin (Figure 3C). Platelet-derived growth factor beta (PDGFRB) filled stromal regions between the glomerular vasculature, further supporting the presence of mesangial cells (Figure 3D). Deposition of a glomerular basement membrane was marked by a pan-laminin stain that localized at the interface between hPODXL and mCD31 (Figure 3E).

DNA$^-$AQP1$^+$ erythrocytes were observed within the glomerular vasculature, demonstrating systemic perfusion and size-selective filtration (Figure 3F). Additional staining with TER119, a specific marker for mouse red blood cells, confirmed their presence within these structures (Figure S3B). Cells within these glomeruli selectively expressed PV1 (PLVAP), a membrane glycoprotein located in the diaphragms of fenestrae of renal endothelial cells[27,28] (Figures 3G and S3C). Transmission electron microscopy (TEM) ultrastructural images of the glomeruli suggested perfusion of red blood cells through capillaries adjacent to presumptive podocytes and mesangial cells and surrounded by parietal epithelial cells (Figure 3H). Low-magnification imaging of the graft with mCD31 showed significant infiltration by host vasculature, which surrounded pockets of human nephron structures, whereas human endothelial cells (hCD31$^+$) were observed within the graft but did not significantly interact with or invade the developing glomeruli (Figure 3I).

As our earlier experiments suggested that NPCs appeared within a few days of CHIR treatment, we tested whether even younger implants (D4) could still produce glomeruli. To generate sufficient cell numbers to form a pellet, the cell seeding density for differentiations was increased 2.5-fold to enable cell pellet formation during the implant-formation process (Figure S3D). D4 grafts consistently formed segmented nephrons *in vivo* similar in appearance and quantity to D7 material tested side by side (Figures 3J and S3E). Compared to D7 grafts, D4 grafts tended to be larger and to contain a higher density of nephron-like structures, including a 2-fold increase in proximal tubular structures that was statistically significant (Figure S3E). Chimeric glomerular structures in D4 grafts were also surrounded by PECs and GATA3$^+$/HNA$^+$ human mesangial cells, but, qualitatively, the glomerulus-like structures in D4 grafts appeared slightly less mature, possibly resembling capillary loop-stage glomeruli (Figures S3F–S3H). After discovering these mesangial cells, we examined the *FOXD1* expression levels in the differentiations by qPCR for additional evidence of stromal progenitor cells in addition to *PDGFRA*.[8] We found a significant increase in signal occurring around D5 compared to D0 (Figure S3I). Together, these data showed that implanted progenitor cells are able to recruit host vasculature to form architecturally complex chimeric glomerular structures.

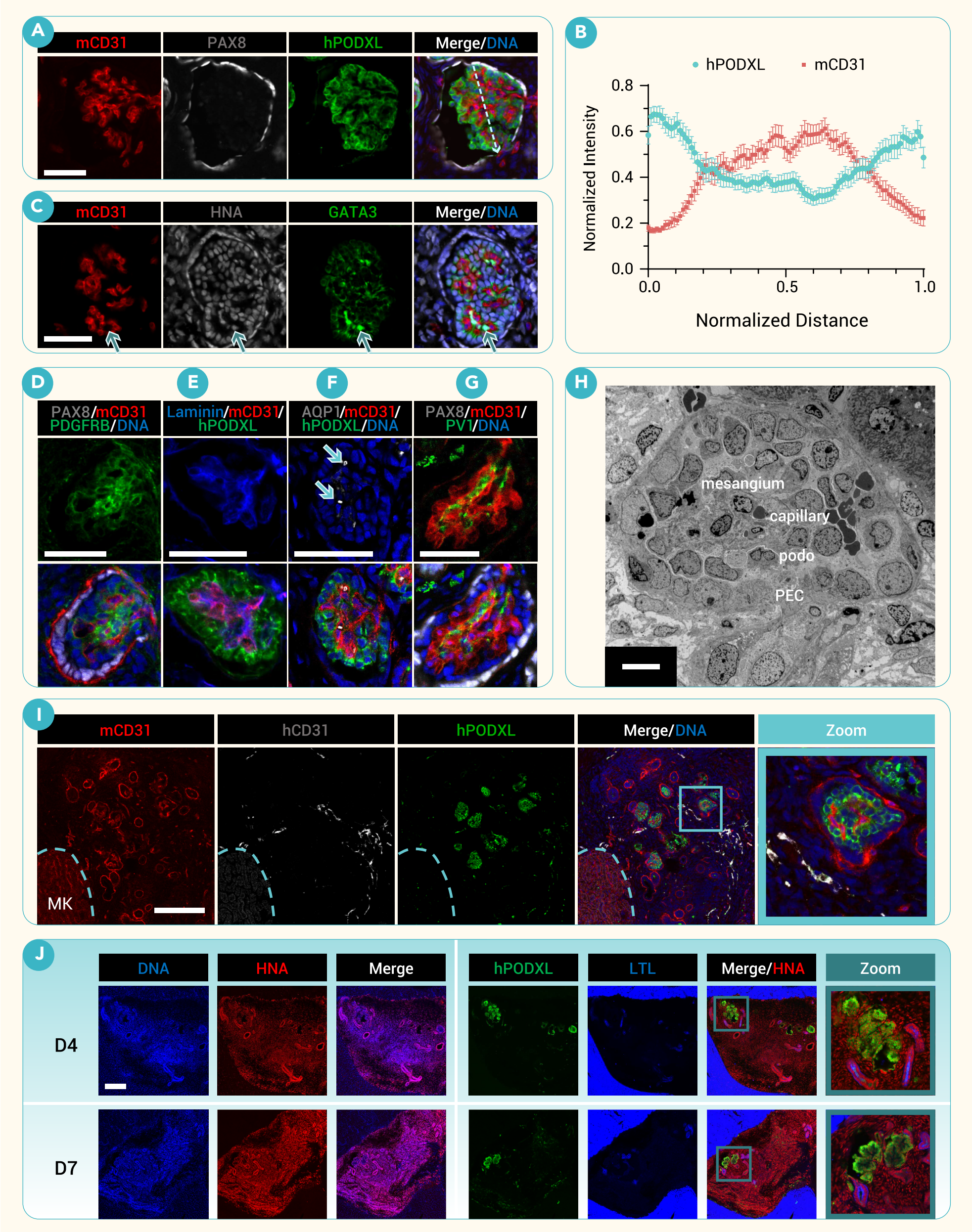

Figure 3. Engrafted human podocytes and mesangial cells recruit mouse endothelial cells to form glomeruli (A) Representative confocal image of a vascularized chimeric glomerulus in the human grafts stained for mouse vasculature (mCD31), human podocytes (hPODXL), and parietal epithelial cells (PAX8). Dashed yellow line depicts an example of how line-scan analysis was performed. (B) Average normalized line scan of fluorescence signal of pooled chimeric glomerular structures (mean ± SEM, n = 25 glomeruli pooled from five D7 grafts). (C) Representative confocal image of GATA3+/HNA+ human mesangial cells (arrow) within chimeric glomerular structures. (D) Representative confocal image of PDGFRβ+ cells within chimeric glomerular structures. (E) Representative confocal image showing the deposition of laminin between hPODXL+ human podocytes and mCD31+ mouse endothelial cells within chimeric glomeruli. (F) Representative confocal image of AQP1+/DNA− erythrocytes (arrows) found within mCD31+ mouse vasculature structures of chimeric glomeruli. (G) Representative confocal image of PV1+ expression within chimeric glomerular structures. (H) Transmission electron microscope (TEM) images of engrafted chimeric glomerular structures. PEC, parietal epithelial cell; podo, podocyte. (I) Confocal immunofluorescent images of mouse (mCD31) and human (hCD31) vasculature and human podocalyxin (hPODXL) staining within the graft and murine kidney (MK). (J) Comparison of representative confocal immunofluorescent images of D4 and D7 grafts after 21 days *in vivo*, containing major nephron segments including podocytes (hPODXL) and proximal tubules (LTL). Scale bars, 50 μm (A, C, and D–G), 8 μm (H), and 200 μm (I and J).

Graft tubules achieve greater maturity than *in vitro* organoids

In addition to glomerular structures, grafts contained major segments of the nephron including proximal and distal tubules, as marked by LTL and epithelial cadherin (ECAD), respectively (Figure 4A). Nephron structures in grafts exhibited specific and statistically significant staining for the proximal tubule marker aquaporin 1 (AQP1), the loop of Henle marker uromodulin (UMOD), and the distal tubule marker GATA3, compared to organoids *in vitro*, in which these markers were largely undetectable (Figures 4B and 4C).[16,29] We note that GATA3 in tubules can be distinguished from its expression in mesangial cells by virtue of cell morphology, location, and co-expressed markers.[30]

Expression of angiotensin-converting enzyme 2 (ACE2), cubilin, and organic anion transporter 1 (OAT1) in proximal tubules was also observed, as was Na-K-Cl cotransporter (NKCC2) and mucin 1 (MUC1) in distal tubules (Figure S4A). Some expression of dolichos biflorus agglutinin (DBA) could be seen, but structures did not express more specific markers of principal and intercalated cells, including aquaporin 4 (AQP4), pendrin (SLC26A4), ETS variant transcription factor 4 (ETV4), and the Ret proto-oncogene (RET), confirming an absence of any ureteric bud or collecting duct segments within the grafts and suggesting that the GATA3 and DBA staining was from connecting tubule-like segments (Figures S4B and S4C).

TEM images of tubules within the graft revealed an abundance of mitochondria and tight junctions located near the apical surface, indicating the potential to carry out energy-intensive transport, but brush borders were not observed (Figure 4D). PV1$^+$/mCD31$^+$ endothelia surrounded PAX8$^+$ tubules, suggesting that fenestrated peritubular capillaries were forming from invading host vasculature and adopting a phenotype specific to the engrafted material (Figure 4E). Myeloid ecotropic insertion site (MEIS1/2/3) and PDGFRB, markers of renal interstitium, were expressed in stromal cells surrounding nephron epithelia (Figures 4F and 4G). Together, these data showed that the engrafted progenitor cells formed segmented nephrons that improved upon the maturation of organoids generated *in vitro*.

DISCUSSION

A detailed study of the optimal stage of differentiation for renal implants derived from iPSCs has not previously been conducted. Our findings indicate that implantation of progenitor cell populations rather than differentiated organoids leads to improved *in vivo* subcapsular engraftment. Remarkably, a mere 4 days of iPSC differentiation *in vitro* is sufficient to specify an iMM population that can proceed to differentiate into sophisticated glomeruli *in vivo*. Conceptually, this finding is reminiscent of earlier observations using primary tissue implants of embryonic metanephroi, which demonstrated superior engraftment when sourced from younger embryos.[31]

In our previous study, we found that mature organoids could survive beneath the capsule and undergo a limited degree of vascularization from the host,[16] whereas, in our study, mature organoids rapidly deteriorated. In the former study, we microdissected the organoids away from the surrounding stroma prior to implantation, whereas, in the present study, we scraped both epithelial structures and stromal cells from the well. It is possible that including the stroma, or alternatively the scraping methodology, contributed to more rapid deterioration. The stroma may provide a substrate onto which mature epithelial

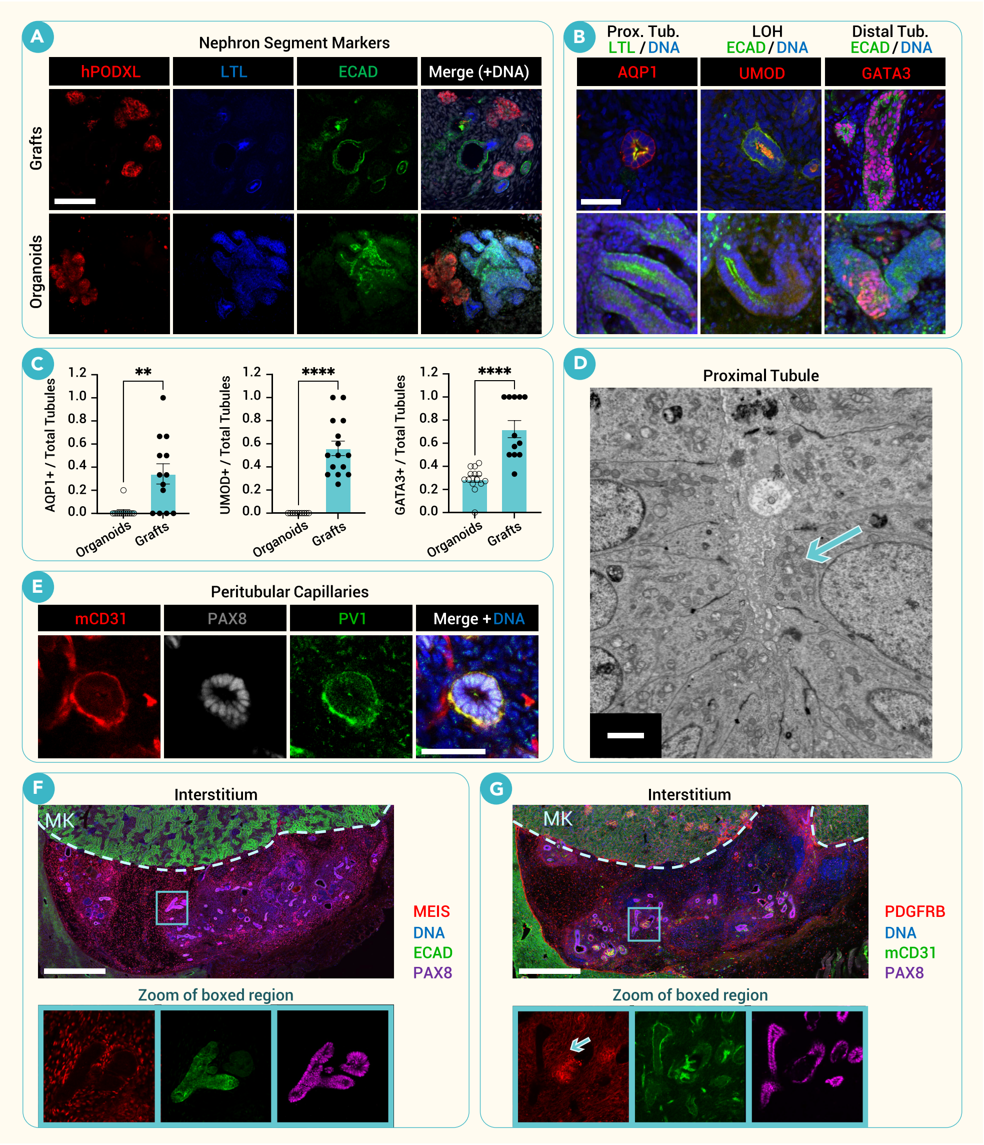

Figure 4. Engrafted tubules achieve greater maturity than *in vitro* organoids (A) Comparison of representative confocal immunofluorescent images of major nephron segments including podocytes (hPODXL), proximal tubules (LTL), and distal tubules (ECAD) within grafts (implanted on day 7 of differentiation *in vitro* and left for 21 days *in vivo*) and organoids (day 21 of differentiation). (B) Immunofluorescent staining for a panel of markers found in proximal tubules (AQP1) with LTL (left), loops of Henle (LOHs) (uromodulin [UMOD]) with ECAD (center) and distal tubules (GATA3) with ECAD (right). (C) Quantification of maturation markers (AQP1, UMOD, and GATA3) in organoids versus grafts shows higher expression in grafts. Staining was scored as the ratio of positive to total structures per image (mean ± SEM, $n \geq 12$ images per condition pooled from four independent experiments; $^{**}p < 0.01$, $^{****}p < 0.0001$ by Welch's t tests). (D) Representative TEM image of a proximal tubule within a graft. Apically located mitochondria marked with arrow. (E) Representative confocal image of $PV1^{+}$ peritubular capillaries. (F and G) (F) Representative immunofluorescent image of interstitial MEIS and (G) PDGFRβ observed in grafts surrounding renal tubules ($PAX8^{+}$) with zoomed channel panels. Arrowhead highlights interstitial signal. MK, mouse kidney cortex. Scale bars, 100 μm (A), 50 μm (B), 2 μm (D), 50 μm (E), and 500 μm (F,G).

cells can outgrow and dedifferentiate, mimicking a process we have observed when organoids are replated *in vitro*.[32] Indeed, cavities that form within grafts of mature organoids are initially surrounded by stromal cells but gradually become lined with $hPODXL^{+}$ cells that appear more epithelial, suggesting an outgrowth process. In contrast, earlier-stage iMM produced more sophisticated glomeruli than mature organoids, even compared to our previous study, because the scraping occurred prior to differentiation of both epithelia and stroma, which were allowed to proceed without interruption *in vivo*.[16] Disordered stroma may also negatively affect vascularization of the graft, driving necrosis.[15,33]

Studies by other groups, utilizing alternative differentiation protocols, have suggested that glomeruli can successfully form from later-stage organoids (day 25).[17] It is possible that other protocols retain NPCs longer than our protocol due to additional treatments with CHIR and growth factors after the initial CHIR pulse. Indeed, one study employed heterochronic mixing of progenitors in an attempt to create a sustained NPC population in mature organoids suitable for long-term engraftment.[19] We intentionally employed a relatively simple differentiation protocol to enable more precise staging of the cultures and define the necessary complexity and time needed to generate successful grafts.

One of the most significant discoveries in this study is that iMM gives rise to human mesangial cells within glomeruli, which are not known to arise in kidney organoids.[14] Mesangial cells, together with podocytes and endothelial cells, are among the major lineages that combine to form glomeruli.[26,34,35] As mesangial cells derive from SPs, the presence of renal mesangium within our grafts implies the existence of an SP population in iMM, which likely arises in sync with the NPCs just as it does *in vivo*.[10] These SPs cannot be readily microdissected from neighboring NPCs, which prevents us from determining definitively which of these populations gives rise to the mesangium. It is possible that other types of renal stromal cells can also differentiate from SPs. Indeed, we and others have observed PDGFRB and MEIS1, markers of renal interstitium, in the abundant stroma that arises within these grafts.[19] In contrast to mesangial cells, however, it is much more difficult to conclusively identify renal interstitium in grafts based on combinations of gene expression markers and morphology. It is now recognized that SPs are critical to renal development, and these are expected to be necessary for successful nephrogenesis from iPSCs.[9,15] The ability to rapidly generate both NPCs and SPs in a single pot, so to speak, greatly simplifies the strategy needed for renal regeneration. This may also improve reproducibility, as variability in organoids may increase with time spent *in vitro*.[11,36]

Capillaries within glomerulus-like structures contain red blood cells, which appear to be filtered by the surrounding podocytes. Additional functional assays, such as size-selective filtration of injected dextrans, require live imaging through abdominal windows but would be interesting to develop in future studies.[37] Despite the impressive integration of iPSC-derived glomeruli with host vasculature, kidney tubules derived from the graft fail to fuse with the host's urinary system, which prevents them from being fully functional. One potential solution to this problem would be to implant progenitor cells within the cortex of the kidney, rather than within the capsule, which would promote interaction between graft tubules and host tubules by virtue of their physical proximity. While we have previously succeeded in implanting organoids into neonatal kidneys,[3] implantation into the adult kidney cortex is technically challenging due to the dense and highly vascularized nature of the tissue and might do more harm than good. Now that we have identified a differentiation stage for implantation, the stage is set to determine which graft site would produce optimal integration, which would be best conducted in a separate follow-up study.

Another significant limitation is that the grafts are still not fully mature and continue to contain an abundance of stromal cells, as previously reported.[16,17,19] These may represent off-target lineages, which naturally arise from iPSCs, reflecting their inherent potential to produce any of the body's numerous cell types.[3,11,13] Further optimization of the differentiation protocol might help eliminate such contaminants.[11] Alternatively, NPCs and SPs in

organoid cultures could be expanded by providing the proper signals for self-renewal *in vitro*, resulting in a scale-up of purified progenitor cells. While such conditions have been described for NPCs from iPSCs, these may select against SPs[20,38]; therefore, it is important to identify conditions that support the expansion of both SPs and NPCs simultaneously. Similarly, it would be beneficial to combine SPs and NPCs with ureteric bud cells, which give rise to the collecting ducts and are required for branching morphogenesis during normal kidney development.[15] We found no evidence for collecting duct differentiation in our grafts, which suggests that ureteric bud is absent in our organoid cultures, even at the earliest stages.[3,6] Careful study of specific progenitor cell populations and renal developmental stages in organoids, together with studies to identify optimal sites for engraftment, may ultimately lead to one-pot recipes of implantable progenitor cells suitable for renal regeneration.

RESOURCE AVAILABILITY

Materials availability

This study did not generate new unique materials/reagents.

Data and code availability

- All datasets, including raw data and statistical analysis, are available upon reasonable request from the corresponding author.

FUNDING AND ACKNOWLEDGEMENTS

The work was supported by Cystinosis Research Foundation grants CRFF-2021–003 and CRFF-2023-004 (B.S.F.), NIH award UC2DK126006 (B.S.F.), a Regular Award from the United States-Israel Binational Science Foundation (B.S.F. and B.D.), an NIH Phase 2 TARGETED Challenge Prize (B.S.F.), the Nowak Macklin Research Fund (B.S.F.), NIH TL1TR002318 (T.V.), and an Institute for Stem Cell and Regenerative Medicine Fellows award (T.V.). The funders had no role in study design, data collection and analysis, decision to publish, or preparation of the manuscript. We thank Ed Parker for assistance with TEM sample processing and imaging. We thank Stuart Shankland, Oliver Wessely, Anton Kary, Aquene Reid, members of the Freedman lab, and members of the NIH ReBuilding a Kidney Consortium for meaningful discussions and feedback. Diagrams and experimental schematics were created using BioRender.

AUTHOR CONTRIBUTIONS

B.S.F. conceived the project with input from T.V. and B.D. T.V., B.D., S.N., O.C.-Z., and B.S.F. designed the experiments. T.V., M.N., O.C.-Z., and S.N. performed the experiments. T.V., M.N., S.N., O.C.-Z., and B.S.F. analyzed the data. T.V., S.N., and B.S.F. wrote the paper with input from all authors. All authors contributed to the manuscript and approved the final version.

DECLARATION OF INTERESTS

B.S.F. is an inventor on patents and/or patent applications related to human kidney organoid differentiation and disease modeling (these include "Three-dimensional differentiation of epiblast spheroids into kidney tubular organoids modeling human microphysiology, toxicology, and morphogenesis" [Japan, US, and Australia], licensed to STEMCELL Technologies; "High-throughput automation of organoids for identifying therapeutic strategies" [PTC patent application pending]; and "Systems and methods for characterizing pathophysiology" [PTC patent application pending]). B.S.F. holds ownership interest in Plurexa LLC. Plurexa was not involved in the research or funding for this study.

SUPPLEMENTAL INFORMATION

It can be found online at https://doi.org/10.1016/j.xinn.2026.101281.

REFERENCES

1. Webster, A.C., Nagler, E.V., Morton, R.L. et al. (2017). Chronic kidney disease. *Lancet* **389**:1238–1252. DOI:10.1016/S0140-6736(16)32064-5
2. Takahashi, K., Tanabe, K., Ohnuki, M. et al. (2007). Induction of pluripotent stem cells from adult human fibroblasts by defined factors. *Cell* **131**:861–872. DOI:10.1016/j.cell.2007.11.019
3. Freedman, B.S., Brooks, C.R., Lam, A.Q. et al. (2015). Modelling kidney disease with CRISPR-mutant kidney organoids derived from human pluripotent epiblast spheroids. *Nat. Commun.* **6**:8715. DOI:10.1038/ncomms9715
4. Morizane, R., Lam, A.Q., Freedman, B.S. et al. (2015). Nephron organoids derived from human pluripotent stem cells model kidney development and injury. *Nat. Biotechnol.* **33**:1193–1200. DOI:10.1038/nbt.3392
5. Takasato, M., Er, P.X., Becroft, M. et al. (2014). Directing human embryonic stem cell differentiation towards a renal lineage generates a self-organizing kidney. *Nat. Cell Biol.* **16**:118–126. DOI:10.1038/ncb2894
6. Taguchi, A., Kaku, Y., Ohmori, T. et al. (2014). Redefining the in vivo origin of metanephric nephron progenitors enables generation of complex kidney structures from pluripotent stem cells. *Cell Stem Cell* **14**:53–67. DOI:10.1016/j.stem.2013.11.010
7. Kobayashi, A., Valerius, M.T., Mugford, J.W. et al. (2008). Six2 defines and regulates a multipotent self-renewing nephron progenitor population throughout mammalian kidney development. *Cell Stem Cell* **3**:169–181. DOI:10.1016/j.stem.2008.05.020
8. Kobayashi, A., Mugford, J.W., Krautzberger, A.M. et al. (2014). Identification of a multipotent self-renewing stromal progenitor population during mammalian kidney organogenesis. *Stem Cell Rep.* **3**:650–662. DOI:10.1016/j.stemcr.2014.08.008
9. Das, A., Tanigawa, S., Karner, C.M. et al. (2013). Stromal-epithelial crosstalk regulates kidney progenitor cell differentiation. *Nat. Cell Biol.* **15**:1035–1044. DOI:10.1038/ncb2828
10. Lindström, N.O., Guo, J., Kim, A.D. et al. (2018). Conserved and Divergent Features of Mesenchymal Progenitor Cell Types within the Cortical Nephrogenic Niche of the Human and Mouse Kidney. *J. Am. Soc. Nephrol.* **29**:806–824. DOI:10.1681/ASN.2017080890
11. Wu, H., Uchimura, K., Donnelly, E.L. et al. (2018). Comparative Analysis and Refinement of Human PSC-Derived Kidney Organoid Differentiation with Single-Cell Transcriptomics. *Cell Stem Cell* **23**:869–881.e8. DOI:10.1016/j.stem.2018.10.010
12. Takasato, M., Er, P.X., Chiu, H.S. et al. (2015). Kidney organoids from human iPS cells contain multiple lineages and model human nephrogenesis. *Nature* **526**:564–568. DOI:10.1038/nature15695
13. Czerniecki, S.M., Cruz, N.M., Harder, J.L. et al. (2018). High-Throughput Screening Enhances Kidney Organoid Differentiation from Human Pluripotent Stem Cells and Enables Automated Multidimensional Phenotyping. *Cell Stem Cell* **22**:929–940.e4. DOI:10.1016/j.stem.2018.04.022
14. Tsujimoto, H., Hoshina, A., Mae, S.I. et al. (2024). Selective induction of human renal interstitial progenitor-like cell lineages from iPSCs reveals development of mesangial and EPO-producing cells. *Cell Rep.* **43**:113602. DOI:10.1016/j.celrep.2023.113602
15. Tanigawa, S., Tanaka, E., Miike, K. et al. (2022). Generation of the organotypic kidney structure by integrating pluripotent stem cell-derived renal stroma. *Nat. Commun.* **13**:611. DOI:10.1038/s41467-022-28226-7
16. Nam, S.A., Seo, E., Kim, J.W. et al. (2019). Graft immaturity and safety concerns in transplanted human kidney organoids. *Exp. Mol. Med.* **51**:1–13. DOI:10.1038/s12276-019-0336-x
17. van den Berg, C.W., Ritsma, L., Avramut, M.C. et al. (2018). Renal Subcapsular Transplantation of PSC-Derived Kidney Organoids Induces Neo-vasculogenesis and Significant Glomerular and Tubular Maturation In Vivo. *Stem Cell Rep.* **10**:751–765. DOI:10.1016/j.stemcr.2018.01.041
18. Sharmin, S., Taguchi, A., Kaku, Y. et al. (2016). Human Induced Pluripotent Stem Cell–Derived Podocytes Mature into Vascularized Glomeruli upon Experimental Transplantation. *J. Am. Soc. Nephrol.* **27**:1778–1791. DOI:10.1681/ASN.2015010096
19. Kumar Gupta, A., Sarkar, P., Wertheim, J.A. et al. (2020). Asynchronous mixing of kidney progenitor cells potentiates nephrogenesis in organoids. *Commun. Biol.* **3**:231. DOI:10.1038/s42003-020-0948-7
20. Tanigawa, S., Naganuma, H., Kaku, Y. et al. (2019). Activin is superior to BMP7 for efficient maintenance of human iPSC-derived nephron progenitors. *Stem Cell Rep.* **13**:322–337. DOI:10.1016/j.stemcr.2019.07.003
21. Namestnikov, M., Cohen-Zontag, O., Omer, D. et al. (2025). Human fetal kidney organoids model early human nephrogenesis and Notch-driven cell fate. *EMBO J.* **44**:4681–4719. DOI:10.1038/s44318-025-00504-2
22. Szot, G.L., Koudria, P. and Bluestone, J.A. (2007). Transplantation of pancreatic islets into the kidney capsule of diabetic mice. *J. Vis. Exp.* **9**:404. DOI:10.3791/404
23. Taylor, S.C., Nadeau, K., Abbasi, M. et al. (2019). The ultimate qPCR experiment: Producing publication quality, reproducible data the first time. *Trends Biotechnol.* **37**:761–774. DOI:10.1016/j.tibtech.2018.12.002
24. Harari-Steinberg, O., Metsuyanim, S., Omer, D. et al. (2013). Identification of human nephron progenitors capable of generation of kidney structures and functional repair of chronic renal disease. *EMBO Mol. Med.* **5**:1556–1568. DOI:10.1002/emmm.201201584
25. Lawlor, K.T., Zappia, L., Lefevre, J. et al. (2019). Nephron progenitor commitment is a stochastic process influenced by cell migration. *eLife* **8**:e41156. DOI:10.7554/eLife.41156
26. Eremina, V., Cui, S., Gerber, H. et al. (2006). Vascular endothelial growth factor a signaling in the podocyte-endothelial compartment is required for mesangial cell migration and survival. *J. Am. Soc. Nephrol.* **17**:724–735. DOI:10.1681/ASN.2005080810
27. Stan, R.V., Kubitza, M. and Palade, G.E. (1999). PV-1 is a component of the fenestral and stomatal diaphragms in fenestrated endothelia. *Proc. Natl. Acad. Sci. USA* **96**:13203–13207. DOI:10.1073/pnas.96.23.13203
28. Wolf, E.E., Steglich, A., Kessel, F. et al. (2023). PLVAP as an early marker of glomerular endothelial damage in mice with diabetic kidney disease. *Int. J. Mol. Sci.* **24**:1094. DOI:10.3390/ijms24021094
29. Helms, L., Marchiano, S., Stanaway, I.B. et al. (2021). Cross-validation of SARS-CoV-2 responses in kidney organoids and clinical populations. *JCI Insight* **6**:e154882. DOI:10.1172/jci.insight.154882
30. Grigorieva, I.V., Oszwald, A., Grigorieva, E.F. et al. (2019). A novel role for GATA3 in mesangial cells in glomerular development and injury. *J. Am. Soc. Nephrol.* **30**:1641–1658. DOI:10.1681/ASN.2018111143
31. Dekel, B., Burakova, T., Arditti, F.D. et al. (2003). Human and porcine early kidney precursors as a new source for transplantation. *Nat. Med.* **9**:53–60. DOI:10.1038/nm812

32. Cruz, N.M., Song, X., Czerniecki, S.M. et al. (2017). Organoid cystogenesis reveals a critical role of microenvironment in human polycystic kidney disease. *Nat. Mater.* **16**:1112–1119. DOI:10.1038/nmat4994
33. Liu, H., Zhang, X., Liu, J. et al. (2023). Vascularization of engineered organoids. *BMEmat* **1**:e12031. DOI:10.1002/bmm2.12031
34. Quaggin, S.E. and Kreidberg, J.A. (2008). Development of the renal glomerulus: good neighbors and good fences. *Development* **135**:609–620. DOI:10.1242/dev.001081
35. Marciano, D.K. (2019). Mesangial cells: The tuft guys of glomerular development. *J. Am. Soc. Nephrol.* **30**:1551–1553. DOI:10.1681/ASN.2019070724
36. Phipson, B., Er, P.X., Combes, A.N. et al. (2019). Evaluation of variability in human kidney organoids. *Nat. Methods* **16**:79–87. DOI:10.1038/s41592-018-0253-2
37. van den Berg, C.W., Koudijs, A., Ritsma, L. et al. (2020). In vivo assessment of size-selective glomerular sieving in transplanted human induced pluripotent stem cell-derived kidney organoids. *J. Am. Soc. Nephrol.* **31**:921–929. DOI:10.1681/ASN.2019060573
38. Huang, B., Zeng, Z., Kim, S. et al. (2024). Long-term expandable mouse and human-induced nephron progenitor cells enable kidney organoid maturation and modeling of plasticity and disease. *Cell Stem Cell* **31**:921–939.e17. DOI:10.1016/j.stem.2024.04.002

The Innovation, Volume *7*

Supplemental Information

Co-induction of stromal and epithelial progenitors for renal regeneration

Thomas Vincent, Samera Nademi, Michael Namestnikov, Osnat Cohen-Zontag, Benjamin Dekel, and Benjamin S. Freedman

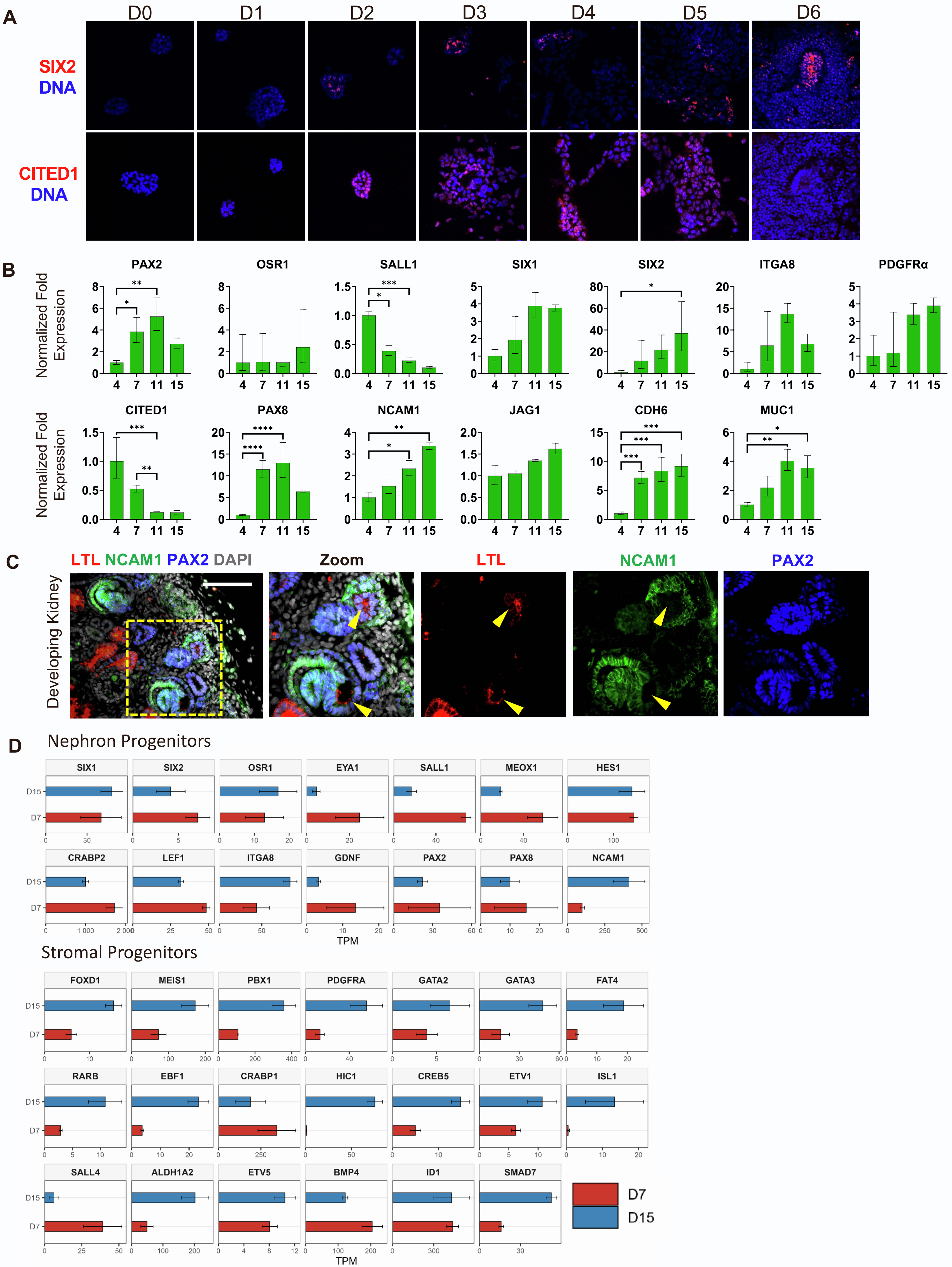
A
D0
D1
D2
D3
D4
D5
D6
SIX2
DNA
CITED1
DNA
B
Normalized Fold Expression
PAX2
OSR1
SALL1
SIX1
SIX2
ITGA8
PDGFRα
CITED1
PAX8
NCAM1
JAG1
CDH6
MUC1
C
LTL NCAM1 PAX2 DAPI
Zoom
LTL
NCAM1
PAX2
Developing Kidney
D
Nephron Progenitors
SIX1
SIX2
OSR1
EYA1
SALL1
MEOX1
HES1
CRABP2
LEF1
ITGA8
GDNF
PAX2
PAX8
NCAM1
TPM
Stromal Progenitors
FOXD1
MEIS1
PBX1
PDGFRA
GATA2
GATA3
FAT4
RARB
EBF1
CRABP1
HIC1
CREB5
ETV1
ISL1
SALL4
ALDH1A2
ETV5
BMP4
ID1
SMAD7
D7
D15
TPM

Supplemental Figure 1. qPCR and immunofluorescence depict temporal changes in cell markers in organoids. (A) Representative confocal immunofluorescence images of organoid differentiations from day 0 to day 6 showing increase in signal of NPC marker, SIX2, and an eventual decrease in CITED1. **(B)** qPCR graphs of commonly used kidney differentiation markers on days 4, 7, 11, and 15 where expression has been reported as a fold change in reference to day 0 (mean ± s.e.m. from n = 3 independent biological replicates, * $p < 0.05$, ** $p < 0.01$, *** $p < 0.001$, **** $p < 0.0001$). **(C)** Representative immunofluorescence image of 20-week-old developing human kidney stained for LTL (red), NCAM1 (green), PAX2 (blue), and DAPI (grey). Differentiating structures express LTL as they lose NCAM1 signal (yellow arrowheads). Scale bars = 100 µm (A, C). **(D)** Bulk RNA-seq gene expression levels of nephron progenitor (NP) and stromal progenitor (SP) markers at day 7 (red) and day 15 (blue) as measured by TPM (transcripts per million, mean ± s.e.m.).

Supplemental Figure 2. Induced metanephric mesenchyme forms superior grafts than differentiated organoids.

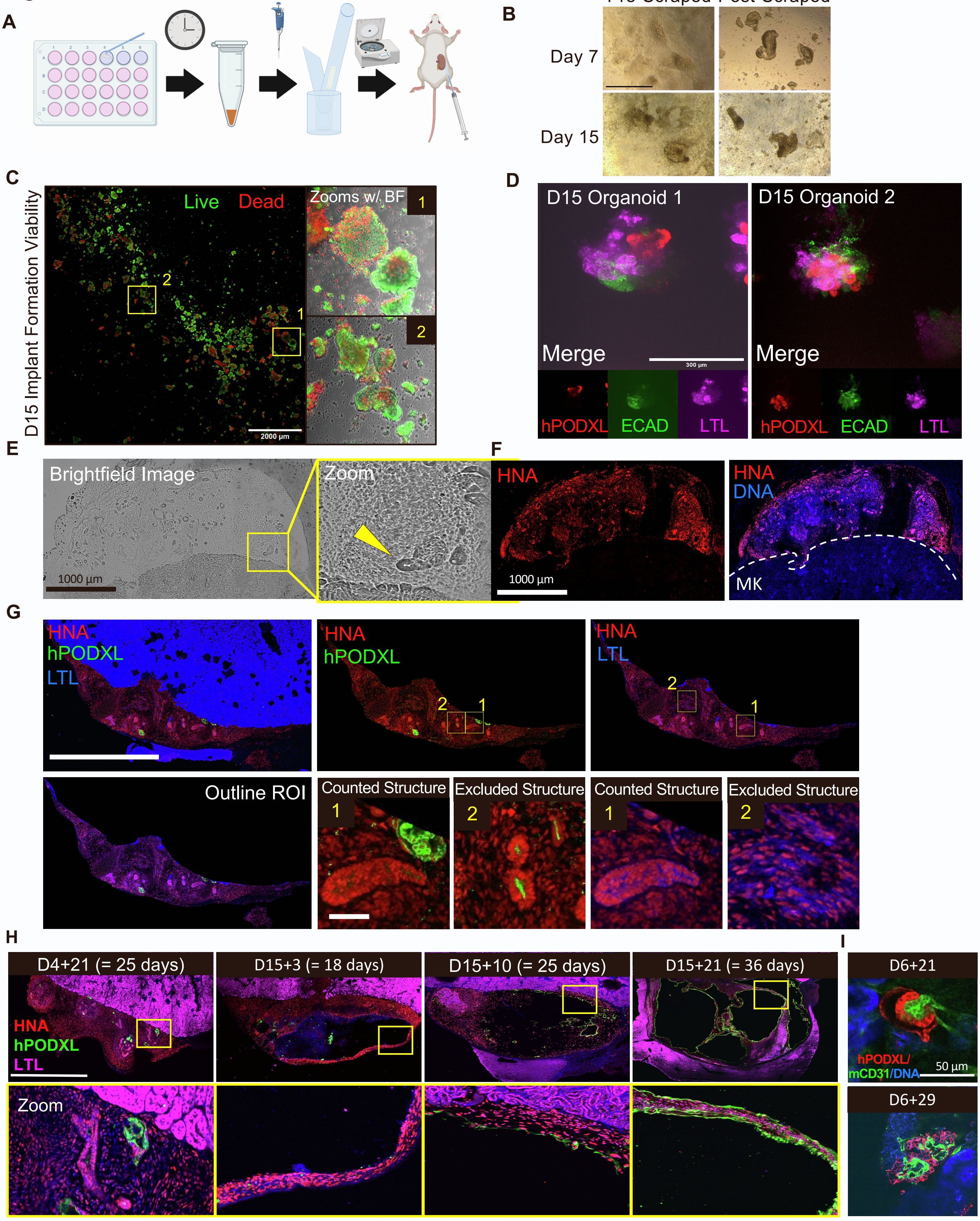

Supplemental Figure 2. Induced metanephric mesenchyme forms superior grafts than differentiated organoids. **(A)** Schematic depicting implant formation process. **(B)** Example brightfield images of day 7 progenitor cell clusters and day 15 organoids pre- and post- manual dissociation from adherent cultures. Scale bar = 1000 µm. **(C)** Viability staining of organoids that have undergone the implant formation process. Live-Dead analysis performed using Calcein AM and propidium iodide. Green cells take in Calcein AM and are viable, red cells have absorbed PI and are dead. Scale bar = 2000 µm. **(D)** Immunofluorescent images of D15 kidney organoids in suspension that have undergone the implant formation process and dispensed into a well for fixation and staining. **(E)** Representative brightfield image of kidney tissue section. Epithelial structures (tubules and glomeruli) appear darker within the grafts (yellow arrow). Scale bar = 1000 µm. **(F)** Representative confocal immunofluorescent image of graft identification using Human Nuclear Antigen (HNA). Dashed white line depicts border between the graft and the host murine kidney (MK) cortex. Scale bar = 1000 µm. **(G)** Representative images depicting typical workflow process for assessing how many PODXL$^+$ and LTL$^+$ structures are present in graft sections representing glomerular and tubule structures respectively. **(H)** Representative confocal immunofluorescence images of early (day 4) and mature (day 15) organoids implanted beneath the kidney capsule for 3, 10, or 21 days. D4 + 21 (25 days) denotes the combined duration of in vitro organoid culture and in vivo implantation, with the total duration provided in parentheses. **(I)** Representative immunofluorescence images of grafts from day 6 organoids implanted under the kidney capsule for 21 or 29 days. Scale bars = 1000 µm and 50 µm for zoomed panels.

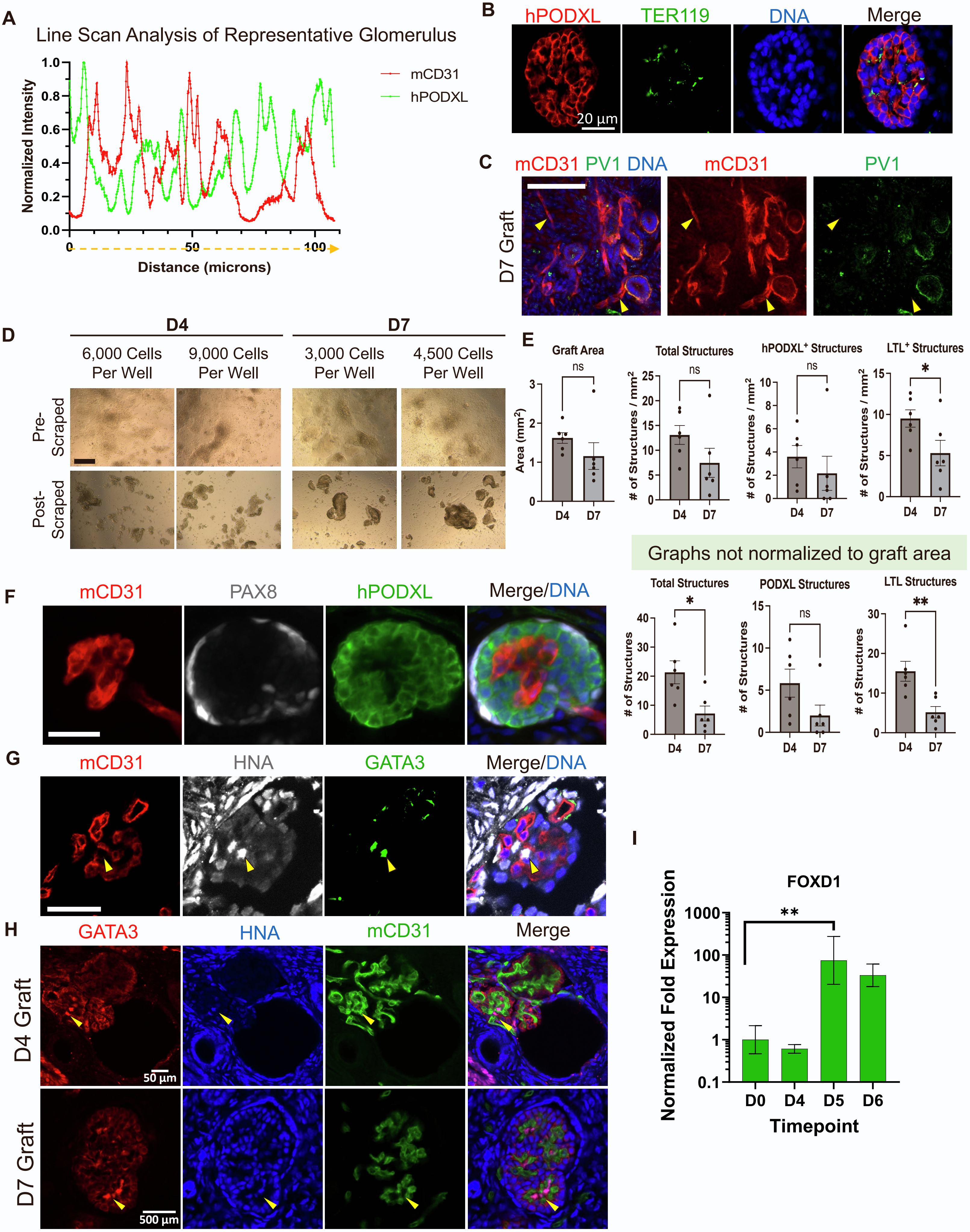
A
Line Scan Analysis of Representative Glomerulus
mCD31
hPODXL
Normalized Intensity
Distance (microns)
B
hPODXL
TER119
DNA
Merge
20 µm
C
mCD31 PV1 DNA
mCD31
PV1
D7 Graft
D
D4
D7
6,000 Cells Per Well
9,000 Cells Per Well
3,000 Cells Per Well
4,500 Cells Per Well
Pre-Scraped
Post-Scraped
E
Graft Area
Total Structures
hPODXL+ Structures
LTL+ Structures
Area (mm2)
# of Structures / mm2
ns
*
Graphs not normalized to graft area
Total Structures
PODXL Structures
LTL Structures
# of Structures
**
F
mCD31
PAX8
hPODXL
Merge/DNA
G
mCD31
HNA
GATA3
Merge/DNA
H
GATA3
HNA
mCD31
Merge
D4 Graft
D7 Graft
50 µm
500 µm
I
FOXD1
Normalized Fold Expression
Timepoint
D0
D4
D5
D6

Supplemental Figure 3. iMM can be implanted at very early timepoints of differentiation. (A) Line scan of fluorescence signal (normalized to the max signal of each channel) of the representative chimeric glomerular structure shown in Figure 3A. **(B)** Representative confocal images showing TER119, a mouse red blood cell marker. $TER119^+$ cells are closely associated with human podocytes ($hPODXL^+$), forming chimeric mouse–human capillary-like structures. $TER119^+$ cells are $DAPI^-$, consistent with their enucleated state (n = 3 mice). **(C)** Confocal image of invading vasculature (mCD31) within the graft showing a lack of PV1 expression (yellow arrows). Scale bar = 100 µm. **(D)** Representative brightfield images of starting cell implant material. Scale bar = 500 µm. **(E)** Quantification of total $hPODXL^+$ and LTL^+ structures in D4 and D7 grafts (p-value = 0.1392) as well as cross sectional area (p-value = 0.2369) of representative graft sections. (mean ± s.e.m. from n = 6 independent implantations per time point) **(F)** Representative confocal immunofluorescent images of D4 grafts containing major components of chimeric glomeruli including human podocytes (hPODXL) and parietal epithelial cells (PAX8) and mouse-derived vasculature (mCD31). Scale bar = 25 µm. **(G)** Representative confocal image of $GATA3^+$/HNA^+ human mesangial cells (yellow arrow) within chimeric glomerular structures. Scale bar = 25 µm. **(H)** Representative confocal images of day 4 and day 7 organoid grafts implanted beneath the kidney capsule for 21 days. **(I)** qPCR graph of *FOXD1* where expression has been reported as a fold change in reference to day 0 (mean ± s.e.m. from n = 3 independent biological replicates, * $p < 0.05$, ** $p < 0.01$, *** $p < 0.001$, **** $p < 0.0001$).

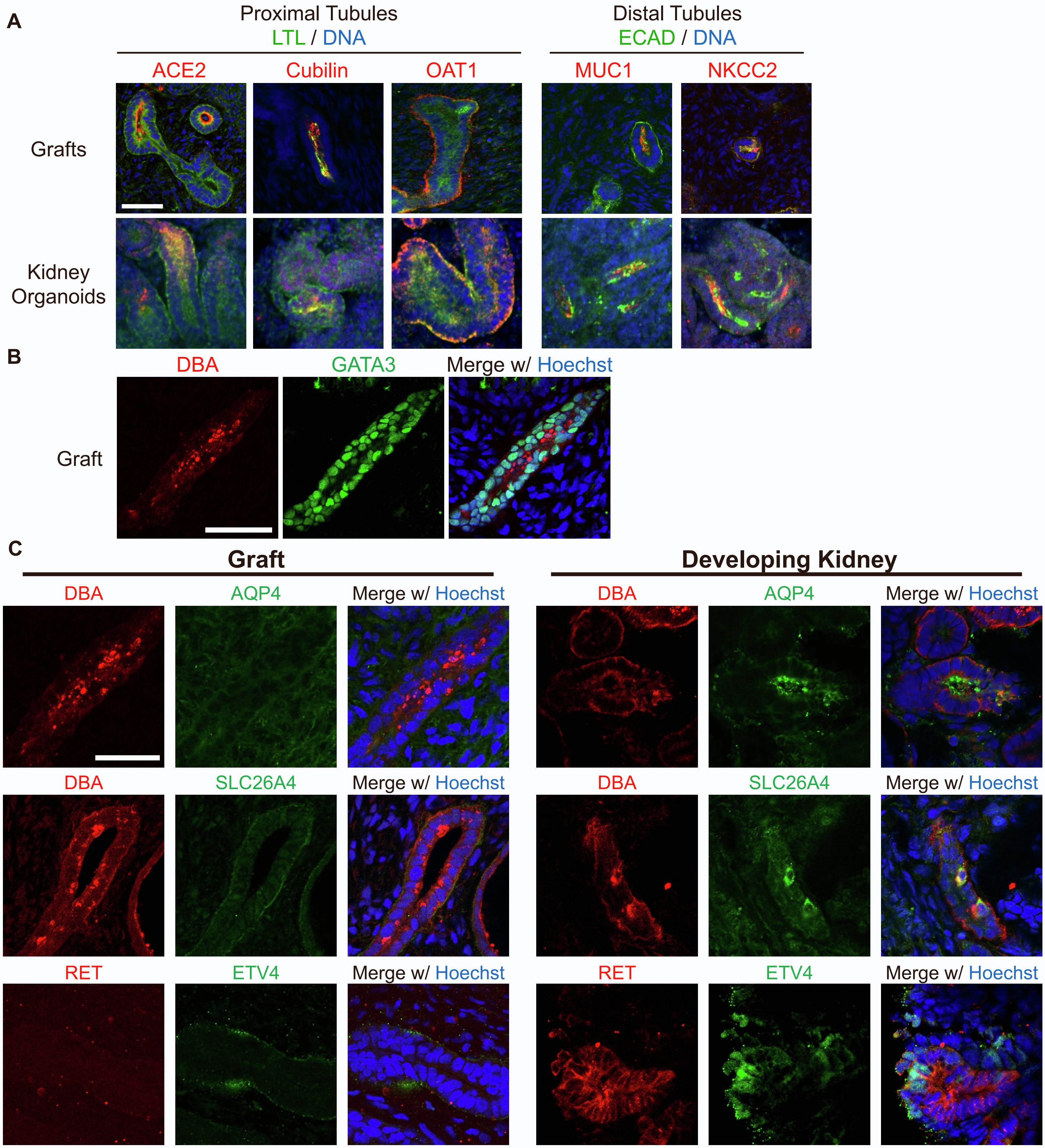

Supplemental Figure 4. Grafts exhibit proximal and distal tubular markers without collecting ducts. (A) Representative immunofluorescent staining in grafts vs. organoids for a panel of markers found in proximal tubules (left, with LTL) and distal tubules (right, with ECAD). (B) Representative immunofluorescent image of graft tubule staining positive for distal markers *Dolichos biflorus* agglutinin (DBA) and GATA3. (C) Representative immunofluorescent images of tubules in grafts (left) and tubules in human developing kidney tissue samples (right) for collecting duct- and ureteric bud-specific markers. Scale bars = 50 μm (A, B) and 25 μm (C).

Supplemental Table 1. List of primers used for RT-qPCR analysis.

Gene	Forward Primer	Reverse Primer
CDH6	CCAGTGGCTCAAACTTTACC	AATGACCACAGGCAAGAGATA
CITED1	AGGATGCCAACCAAGAGATG	GTTTAGTGGGAGGGGTGGTT
CUBN	AATGGATGTGTGCAGCTCAG	GGGGTTGCTCAAACACTCAT
EPCAM	TCTCATCGCAGTCAGGATCATAA	AATCGTCAATGCCAGTGTACTT
FOXD1	GCTCGAGGAAGAAGGTAGGAATC	GCATAGGTCGGCTTTGCATAAAT
HNF1β	GCGCTCAACACCGAGGAG	CAGCAGCTGATCCTGACTGC
ITGA8	GTGCAGTGGGACGACTCGAA	ACACCAGGGATGCAAGAGCATAG
JAG1	TGCCAAGTGCCAGGAAGT	GCCCCATCTGGTATCACACT
LHX1	GGAGCGAAGGATGAAACAGC	TGCGGGAAGAAGTCGTAGTT
LRP2	GCAGACCTAAAGGAGCGTTCG	ATGCGCACTGTCACATTCTTG
MUC1	TCCTCACAGTGCTTACAGTTGTT	ACAGGAAAAAGAAAGAGACCCCA
NCAM1	AACAAAGCATGATGGGTGAA	GTCTGTGGTGTTGGAAATGC
NPHS1	GATCCTCTCCATCCTGGTTCCT	CTCAGGAGAATGGTGATGTCAGG
OSR1	GACATCTGCCACAAAGCCTTC	CCCACAGGTTCTATTTAGCATTTGA
PAX2	AACGACAGAACCCGACTATG	ATCCCACTGGGTCATTGGAG
PAX8	CAAGGTGGTGGAGAAGATTG	GAGGTTGAATGGTTGCTG
PDGFRα	GTGCGAAGACTGAGCCAGATTG	CGATAAACAGAATGCTTGAGCTGTG
PODXL	CAGACCGTGGTCGTCAAAGA	TTCATGTCACTGACCCCTGC
SALL1	TGCAAACAGAATGCAAGCGTTA	AGGCATTGCATCAACACCAGA
SIX1	GGTTTAAGAACCGGAGGCAAAG	AGGACCGAGTTCTGGTCTGGA
SIX2	ACAGGTCAGCAACTGGTTCAAGA	ACTTGCCGCTGCCATTCA

Supplemental Table 2. List of antibodies used for immunofluorescence analysis.

Antibody	Source	Reference	Dilution
ACE2	R&D Systems	Cat. No. AF933	1:40
AQP1	MilliporeSigma	Cat. No. AB2219	1:250
AQP4	Santa Cruz Biotechnology	Cat. No. sc-32739	1:50
hCD31-PE	Miltenyi Biotech	Cat. No. 130-117-225	1:500
mCD31	BD Biosciences	Cat. No. 557355	1:100
CITED1	Invitrogen	Cat. No. PA5-65541	1:300
Cubilin	Abcam	Cat. No. 191073	1:500
DBA	Vector Labs	Cat. No. RL-1032	1:200
ECAD	Abcam	Cat. No. ab11512	1:300
ETV4	ProteinTech	Cat. No. 10684-1-AP	1:100
GATA3	R&D Systems	Cat. No. AF2605	1:100
HNA	EMD Millipore	Cat. No. MAB1281	1:300
Pan-Laminin	Abcam	Cat. No. ab11575	1:300
LTL	Vector Labs	Cat. No. B-1325	1:500
MEIS 1/2/3	Active Motif	Cat. No. 39795	1:1000
MUC1	Abcam	Cat. No. ab15481	1:200
NCAM	Santa Cruz	Cat. No. sc-106	1:250
NKCC2	ProteinTech	Cat. No. 18970-1-AP	1:500
OAT1	Invitrogen	Cat. No. PA5-26244	1:500
PAX2	BioLegend	Cat. No. 901001	1:250
PAX8	ProteinTech	Cat. No. 10336-1-AP	1:100
PDGFRβ	R&D Systems	Cat. No. AF385	1:100
PV1	Abcam	Cat. No. ab81719	1:100
hPODXL	R&D Systems	Cat. No. AF1658	1:500
RET	R&D Systems	Cat. No. AF1485	1:300
SIX1	Cell Signaling Technology	Cat. No. 12891	1:1000
SIX2	ProteinTech	Cat. No. 11562-1-AP	1:300
SLC26A4	Sigma	Cat. No. SAB2104723	1:500
UMOD	Sigma	Cat. No. SAB1400296	1:100
